# The Cytotoxicity of Elderberry Ribosome-Inactivating Proteins Is Not Solely Determined by Their Protein Translation Inhibition Activity

**DOI:** 10.1371/journal.pone.0132389

**Published:** 2015-07-06

**Authors:** Chenjing Shang, Qiushi Chen, Anne Dell, Stuart M. Haslam, Winnok H. De Vos, Els J. M. Van Damme

**Affiliations:** 1 Department of Molecular Biotechnology, Faculty of Bioscience Engineering, Ghent University, Ghent, Belgium; 2 Department of Life Sciences, Faculty of Natural Sciences, Imperial College London, South Kensington Campus, London, United Kingdom; 3 Department Veterinary Sciences, Faculty of Pharmaceutical, Biomedical and Veterinary Sciences, Antwerp, Belgium; New York State Dept. Health, UNITED STATES

## Abstract

Although the protein translation inhibition activity of ribosome inactivating proteins (RIPs) is well documented, little is known about the contribution of the lectin chain to the biological activity of these proteins. In this study, we compared the *in vitro* and intracellular activity of several *S*. *nigra* (elderberry) RIPs and non-RIP lectins. Our data demonstrate that RIPs from elderberry are much more toxic to HeLa cells than to primary fibroblasts. Differences in the cytotoxicity between the elderberry proteins correlated with differences in glycan specificity of their lectin domain, cellular uptake efficiency and intracellular destination. Despite the fact that the bulk of the RIPs accumulated in the lysosomes and partly in the Golgi apparatus, we could demonstrate effective inhibition of protein synthesis *in cellula*. As we also observed cytotoxicity for non-RIP lectins, it is clear that the lectin chain triggers additional pathways heralding cell death. Our data suggest that one of these pathways involves the induction of autophagy.

## Introduction

Plant ribosome-inactivating proteins (RIPs) possess highly specific rRNA *N*-glycosidase activity and are capable of catalytically inactivating eukaryotic ribosomes. This inactivation occurs through the removal of a specific adenine residue from a highly conserved (sarcin/ricin) loop of the large ribosomal RNA [1,2]. Based on their domain architecture, RIPs can be divided into two main categories. Type 1 RIPs such as saporin and trichosanthin represent a group of single-chain proteins with enzymatic activity. The type 2 RIPs are chimeric proteins composed of an A-chain with protein synthesis inhibition activity and a B-chain with carbohydrate-binding/lectin activity [3]. Infamous type 2 RIPs are ricin and abrin, potent toxins that are present in the seeds from *Ricinus communis* (castor bean) and *Abrus precatorius* (jequirity bean), respectively. Some well-known type 2 RIPs (such as ricin, abrin and volkensin) have been shown to exert anti-tumor activity [4]. This has sparked interest in their use for potential therapeutic applications. However, there are major differences between the cytotoxicity of different type 2 RIPs, and while some type 2 RIPs (e.g. from *Sambucus nigra* [5]) exhibit strong protein synthesis inhibition activity *in vitro*, they can be as much as 10^3^−10^5^ less toxic than ricin when administered to animal cells. Possible explanations for this discrepancy include differential cellular uptake efficiency and/or intracellular trafficking of the proteins. It has been shown that the cytotoxicity of certain type 2 RIPs relies on the binding of the B-chain to glycoconjugates at the cell surface, as such facilitating cellular uptake of the ribosome-inactivating proteins [6]. Incubation of mammalian cells with ricin results in its endocytosis and subsequent transport to the trans-Golgi network, followed by retrograde transport from the Golgi apparatus to the ER, where the disulphide bridge between the A- and B-chain is cleaved. Finally, the A-chain enters the cytoplasm where it exerts its N-glycosidase activity [7–9]. Volkensin, follows a similar internalisation pathway as ricin [10]. In contrast, nigrin b, which has comparable plasma membrane binding affinity to volkensin (approx. 10^-10^M), enters the cytosol without passing the trans-Golgi network and ER [10–12]. These data show that the intracellular trafficking pathway (co-)determines cytotoxicity. However, for many RIPs, information on the internalisation kinetics is lacking or incomplete.

As for their cytotoxic activity, it has been reported that RIPs induce apoptotic cell death through different mechanisms, often involving the induction of the unfolded protein response and mitochondrial dysfunction [13–15]. One of the pending questions is whether protein synthesis inhibition is the sole responsible for this RIP-induced apoptosis. The contribution of a lectin domain could modulate RIP activity *in vivo*, either in an antagonistic or synergistic manner. Providing support for the latter, various lectins have been shown to induce cytotoxicity [16,17].

Studies to evaluate the cytotoxicity and/or internalization of type 2 RIPs have mainly focused on proteins with similar, fairly generic carbohydrate recognition domains (mostly galactose-binding) [10,14,18–20]. However, other specific sugar-binding domains may bear more potential for selective targeting of certain (e.g. cancer) cell types. Elderberry (*Sambucus nigra*) produces several type 2 RIPs (SNA-I, SNA-V and SNLRP) and lectins (SNA-II and SNA-IV), with a variety of carbohydrate binding properties, which form an ideal model system to investigate their differential cytotoxicity towards mammalian cells. Glycan array analyses revealed that SNA-I shows strong binding to Neu5Ac(α2–6)Gal/GalNAc, while SNA-II (corresponding to the lectin domain of SNA-V), SNA-IV and SNA-V exhibit clear interaction with Gal/GalNAc residues. Furthermore, SNA-IV is able to recognize Gal residues in sialylated complex glycans occurring in the α2–6 as well as the α2–3 linkage. In contrast to the other *S*. *nigra* proteins, which interact with terminal residues from glycans, SNLRP recognizes the core N-glycan structure since it shows reactivity towards GlcNAc oligomers as well as N-glycans [21]. At present, mostly SNA-I and SNA-V have been studied for their biological properties [21–25].

Since all type 2 RIPs show a clear protein translation inhibition activity *in vitro* [22,26], but exhibit clear differences in cytotoxicity, it is conceivable that the B-chain has an important modulatory role. However, at present the exact working mechanisms are unresolved. To enhance insight in the mode of action of the different *S*. *nigra* type 2 RIPs (SNA-I, SNA-V and SNLRP) and non-RIP lectins (SNA-II and SNA-IV), we investigated their behaviour *in vitro* and *in cellula*. Our data confirmed that the elderberry RIPs are much less toxic than the classical RIPs from *Ricinus* and *Abrus*, but also showed that mortal cell cultures (fibroblasts) were less susceptible to the elderberry proteins than HeLa cell lines. In addition, our results revealed that differences among the elderberry proteins correlate with uptake efficiency and the glycan specificity of their lectin domains.

## Materials and Methods

### RIPs and lectins

All proteins from *Sambucus nigra* were purified by affinity chromatography and gel filtration as described previously [27–30]. SNA-I, SNA-II, SNA-V and SNLRP were isolated from lyophilized *S*. *nigra* bark and SNA-IV from fruits. *S*. *nigra* RIPs (SNA-I, SNA-V and SNLRP) were reduced by incubation with 0.025 M dithiothreitol (DTT) at 37°C for 1 h as described by Emmanuel et al. [31].

### Cell culture

HeLa (Cervix carcinoma, American Type Culture Collection, Manassas, Virginia, USA), NHDF (human dermal fibroblasts, passage 9, PromoCell GmbH, Heidelberg, Germany) and Luc2-IRES-tCD cell cultures (a gift from Dr. Pierre Busson group, Institut de Cancérologie Gustave Roussy, Villejuif, France) [32] were grown in advanced DMEM (Life Technologies, Merelbeke, Gent) supplemented with 2% fetal calf serum (Life Technologies) and 1% L-glutamine Penicillin-Streptomycin-Glutamin solution (Life Technologies) in an incubator set at 37°C and 5% CO_2_.

### Protein synthesis inhibition activity

Protein synthesis inhibition activity for non-reduced or reduced *S*. *nigra* RIPs (SNA-I, SNA-V or SNLRP) was determined using the TnT T7 Quick Coupled Transcription/Translation System Kit (Promega, Mannheim, Germany) based on a cell-free system [20]. The lectins SNA-II and SNA-IV were also included in this assay. According to manufacturer’s instructions, the prepared mixture was incubated at 30°C for 10 min and chilled on ice. Afterwards, 2 μl PBS or PBS containing different concentrations of *S*. *nigra* RIPs or lectins were added to the reaction mixture and incubated for 30 min at 30°C. After addition of 35 μl nuclease-free water at room temperature the reaction samples were transferred to a luminometer plate (Greiner Labortechnik, Frickenhausen, Germany) containing 5 μl luciferase assay reagent at 25°C. The relative luciferase activities of the samples were determined at 562 nm for 10 sec using a microtiter top plate reader (Infinite 200, Tecan, Mannedorf, Switzerland) with an initial delay of 2 sec.

### Cytotoxicity assay

To study the effect of different *S*. *nigra* RIPs and lectins on cell viability and proliferation, a total of 3,000 HeLa or NHDF cells were seeded in a 96-well plate (Greiner Labortechnik) and incubated at 37°C and 5% CO_2_ for 24 h. Subsequently, the medium was exchanged with medium supplemented with various concentrations of *S*. *nigra* RIPs or lectins (ranging from 0.1 to 2 μM), and incubated at 37°C and 5% CO_2_ for 2 time points (24 h and 48 h), respectively. Phosphate buffered saline (Life Technologies) with/without 2 μM BSA was used in the control treatments [33]. Four technical replicates were performed for each concentration, and each experiment was repeated three times.

Cell viability was determined by means of (resazurin-based) Presto Blue spectrophotometric assays (Life Technologies) according to manufacturer’s instructions. In brief, the culture medium of each well was replaced with fresh culture medium containing 10% final concentration of Presto blue reagent. After incubation for 20 min in the dark at 37°C and 5% CO_2_ the fluorescence intensity of reduced resazurin was measured at 560/600 nm in a plate reader (Infinite 200, Tecan, Mannedorf, Switzerland).

Cell morphology was assessed using an inverted transmitted light microscope (Ti Eclipse, Nikon Instruments, Paris, France), with a 10x dry objective (Numerical aperture 0.5).

### Labelling of *S*. *nigra* RIPs and lectins with fluorescein isothiocyanate

*S*. *nigra* RIPs and lectins were labeled with fluorescein isothiocyanate (FITC) (Sigma-Aldrich, St. Louis, USA). Lyophilized protein (10 mg/ml) was dissolved in 0.1 M carbonate/bicarbonate (1:9) buffer, pH 9. Afterwards, a 24-fold molar excess of FITC in dimethylformamide was added to the protein. After 2 h incubation in the dark at room temperature, the labeled protein was purified by gel filtration on a Sephadex G25 column (1 cm diameter/ 5 cm height) using PBS as running buffer. Subsequently, the labeled protein fraction was analyzed by SDS-PAGE and visualized by Fujifilm FLA 5100 (FUJIFILM Life Science, Japan). Protein concentrations and molar FITC/protein ratios were calculated to estimate the labeling efficiency according to manufacturer’s instructions (Thermo scientific, Rockford, USA).

### (Immuno-) fluorescence staining

HeLa cells were seeded on coverslips in a 12 well plate (3.8 × 10^4^ cells/well) for at least 24 h, and cells were incubated with culture medium containing 50 nM FITC-labeled or non-labeled *S*. *nigra* proteins for fixed time periods. After washing with PBS, cells were fixed with 2% formaldehyde for 20 min, followed by three more PBS washes. Subsequently, cells were permeabilized in 0.5% Triton X-100 solution for 5 min, blocked with 50% fetal calf serum for 40 min at room temperature and incubated for 1 h at room temperature with one of the following primary antibodies: mouse anti-PDI (1:1000, Endoplasmic reticulum marker), anti-Golgin97 (1:2000, Golgi marker), rabbit anti-Rab5 (1:1000, Early endosome marker) or p62/SQSTM1 (1:100, autophagic flux marker, Santa Cruz Biotechnology Inc. Texas, USA). After three washes with PBS, cells were incubated for 1 h with goat anti-mouse IgG or goat anti-rabbit IgG Alexa Fluor-555 and after a final wash counterstained with DAPI (0.1 μg/ ml) and mounted with Vectashield (Vector Laboratories Inc., Burlingame, CA, USA).

Next to the immunostaining, lysosomes were visualized with LysoTracker (Life technologies, 50 nM) and lipid droplets were stained with BODIPY 493/503 (Life Technologies, 2 μg/ml).

### *In cellula* luciferase assay

To study translation inhibition *in cellula*, a total of 3,000 HeLa cells (HG1-luc2-IRES-tCD cells [20]) were seeded per well in a 96-well plate (Greiner) and incubated at 37°C and 5% CO_2_ for 24 h. After replacing the medium, the cells were exposed to medium supplemented with 100 nM *S*. *nigra* RIPs and SNA-II, and incubated at 37°C and 5% CO_2_ for another 24 h. Cycloheximide (10 μg/ml, Sigma-Aldrich) was used as the control treatment [33]. Luciferase activity in cell extracts was assessed using the Promega Luciferase Assay System according to the manufacturer’s instructions (Promega, Mannheim, Germany). After washing with PBS, cells were lysed with lysis buffer (Promega luciferase kit) for 2 min and half of the cell lysate was transferred to the luminometer plate (Greiner). After addition of the D-Luciferin substrate, luminescence was measured for a period of 10 seconds. Recorded signals were normalized to the amount of viable cells as measured in a subsequent Presto blue assay. Four technical replicates were performed for each protein concentration, and each experiment was repeated three times.

### Autophagic flux assay

The ptfLC3 expression vector from Dr. Tamotsu Yoshimori encoding an mRFP-EGFP-LC3 fusion construct [34] was purchased from Addgene (Cambridge, MA USA, plasmid 21074). The expression vector was introduced into HeLa cells using Lipofectamine 2000 (Life Technologies) according to the manufacturer’s instructions. One day after transfection, the transfected cells were incubated with 100 nM SNA-I for 7 h. Subsequently the cells were analysed by confocal microscopy.

### Microscopy and image analysis

Confocal images were acquired with a Nikon A1R confocal system, mounted on a Nikon Ti microscope body using a 40x (Numerical aperture 1.3) oil or 60x (Numerical aperture 1.4) oil objective and appropriate filters. The amount of internalized FITC-labeled *S*. *nigra* protein was quantified by measuring the total signal intensity per cell using a home-written script for FIJI image analysis freeware (http://fiji.sc/Fiji). More than 60 cells were analyzed for each treatment, and three repeats were performed for each experiment. The fluorescence signal data was normalized by the FITC labeling efficiency of the *S*. *nigra* proteins. Colocalization analysis was performed making use of the JaCoP plugin (Just Another Co-localization Plugin). Specifically, Manders’ coefficients were retrieved for a fixed threshold setting per dye combination [35] and object-based colocalization was calculated using the centre-of-mass method [36]. An alternative object-based colocalization analysis was also performed using an in-house developed method that calculates the overlap of binarized channels per object [37].

For quantification of p62 foci or BODIPY stained lipid-droplets, images were captured by widefield fluorescence microscopy (Nikon Ti) using a 40x oil objective (Numerical aperture 1.3). At least 25 randomly selected areas in each sample (3 repeats) were recorded using fixed acquisition settings. Per cell, metrics such as mean signal intensity, spot number, and mean spot intensity were measured using an in-house developed cytometric analysis pipeline for FIJI (InSCyDe [38]).

### Mass spectrometry

To acquire N- and O-glycans from glycoproteins, HeLa and NHDF samples were treated following an established protocol [39,40]. Briefly, cells were suspended in lysis buffer (25 mM Tris, 150 mM NaCl, 5 mM EDTA and 1% CHAPS (v/v), pH 7.4) before homogenisation and sonication were performed. The homogenates were subsequently dialysed against a 50 mM ammonia bicarbonate buffer, pH 7.5, after which the samples were lyophilized. Extracted glycoproteins were reduced, carboxymethylated and tryptic digested prior to the release of protein linked N-glycans by PNGase F (Roche Applied Science) digest and O-linked glycans by reductive elimination. Released N- and O-glycans were permethylated prior to MS analysis. Sialidase cleavage was carried out using Sialidase S (Prozyme Glyko) and Sialidase A (Prozyme Glyko) in 50 mM sodium acetate, pH 5.5.

To acquire glycans from glycolipids another protocol was used as described previously [41]. Briefly, the HeLa and NHDF samples were sonicated in ice-cold ultra-pure water. Glycolipids were extracted by chloroform/methanol/water, followed by the release of lipid-linked glycans via rEGCase II (Takara) digestion, and then the glycan purification using a Sep-pack C18 cartridge (Waters) and subsequently a Hypercarb column (Thermo Scientific). After this a deuteroreduction step was carried out. Sialic acids were cleaved using Sialidase S (Prozyme Glyko) and Sialidase A (Prozyme Glyko) in 50 mM sodium acetate, pH 5.5. The treated samples were lyophilized, permethylated and purified using Sep-Pak (C18; Waters).

MS data were obtained via a Voyager MALDI-TOF (Applied Biosystems) mass spectrometer. Purified permethylated glycans were dissolved in 10 μl methanol and 1 μl of the sample was mixed with 1 μl of matrix, 20 mg/ml 2,5-dihydroxybenzoic acid (DHB) in 70% (v/v) aqueous methanol and loaded on to a metal target plate. The instrument was run in the reflectron positive ion mode using an accelerating voltage of 20 kV.

MS/MS data were acquired using a 4800 MALDI-TOF/TOF mass spectrometer (AB SCIEX). In the MS/MS experiment the dissolved sample was dried and then re-dissolved in 10 μl methanol, 1 μl of the sample was mixed with 1 μl of matrix, 10 mg/ml diaminobenzophenone (DABP) in 70% (v/v) aqueous acetonitrile and loaded on to a metal target plate. The instrument was run in the reflectron positive ion mode. The collision energy was set to 1 kV with argon as the collision gas. The 4700 calibration standard (mass standards kit for the 4700 proteomics analyzer, Applied Biosystems) was used as the external calibrant for the MS and MS/MS modes.

The MS and MS/MS data were processed employing Data Explorer Software from Applied Biosystems. The processed spectra were annotated using a glycobioinformatics tool, GlycoWorkBench [42]. Based on known biosynthetic pathways and susceptibility to PNGase F digestion, reductive elimination and rEGCase II digestion, all N-glycans are presumed to have a Manα1–6(Manα1–3)Manβ1–4GlcNAcβ1–4GlcNAc core structure; all O-glycans are assumed to have reducing end GalNAc; all glycolipid derived glycans are assumed to have a core of Galβ1-4Glc [40,43,44]. The symbolic nomenclature used in the spectra annotation is the same as the one used by the Consortium for Functional Glycomics (CFG) (http://www.functionalglycomics.org/) and the Essentials for Glycobiology on-line textbook (http://www.ncbi.nlm.nih.gov/books/NBK1931/Fig/ch1.f5/?report=objectonl).

### Statistical analyses

Statistical analyses were performed using Prism version 5 (GraphPad, La Jolla, CA). One-way ANOVA (Dunnett’s Multiple Comparison Test) was performed at the level of significance (*: p < 0,05; **: p< 0,01; ***: p < 0,001). The 50% lethal concentration (LC50) was determined using the non-linear regression analysis. Data were represented as means ± standard error (SE). Differences in cytotoxicity between different *S*. *nigra* proteins were statistically analyzed using SPSS (Tuckey’s b)—IBM Statistical Package for the Social Sciences Statistics (IBM SPSS Statistics, IBM, New York, US).

## Results

### Elderberry RIPs inhibit protein synthesis *in vitro*

**Fig 1:**
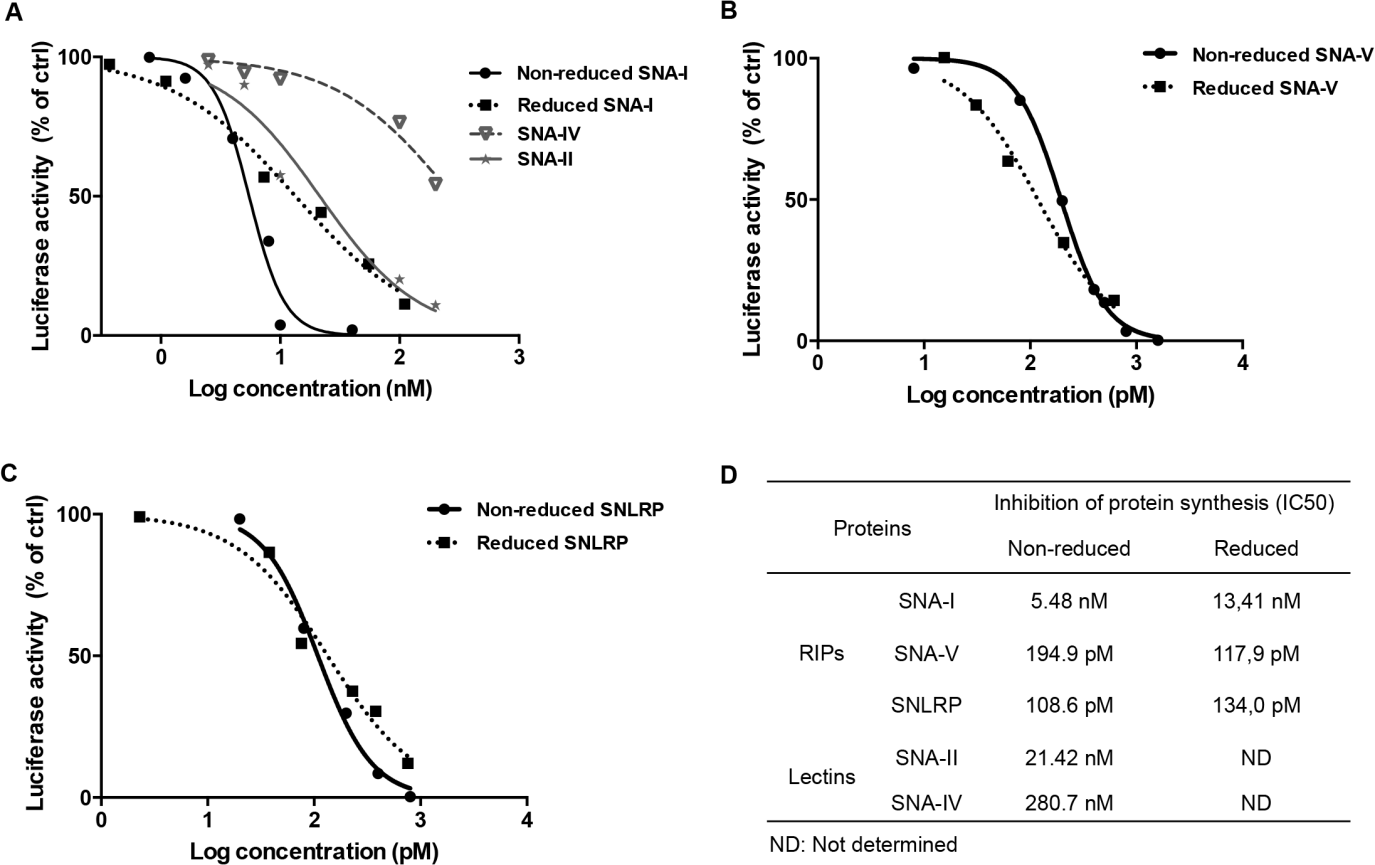
Effect of the *S*. *nigra* RIPs and lectins on protein synthesis in a cell-free translation assay. (A) Dose response curves of luciferase synthesis in treatments with SNA-I (non-reduced and reduced) and lectins (SNA-II, SNA-IV). (B and C) Dose response curves of luciferase synthesis in the treatments with non-reduced and reduced RIP for SNA-V and SNLRP, respectively. (D) IC50 values for the RIPs and lectins.

In an *in vitro* cell-free system, all non-reduced and reduced RIPs (SNA-I, SNA-V and SNLRP) from *S*. *nigra* clearly showed a concentration-dependent translation-inhibiting activity, albeit with strongly varying potency (Fig 1A and 1B) (S1 Fig). Compared to SNA-I (IC50 = 5.48 nM), the IC50 values for *S*. *nigra* RIPs SNA-V and SNLRP were significantly lower (25 to 50 fold) (Fig 1D), indicating that their activity was proportionally higher. The lectins SNA-II and SNA-IV, which are merely composed of the lectin chain, also started interfering with the translation process in the dose range of SNA-I (Fig 1A). The protein synthesis inhibition activity was not enhanced by DTT-mediated reduction of the proteins since the IC50 values for the non-reduced and reduced SNA-I, SNA-V and SNLRP are very similar (Fig 1).

### *S*. *nigra* RIPs and lectins are more toxic towards HeLa than NHDF cells

**Fig 2:**
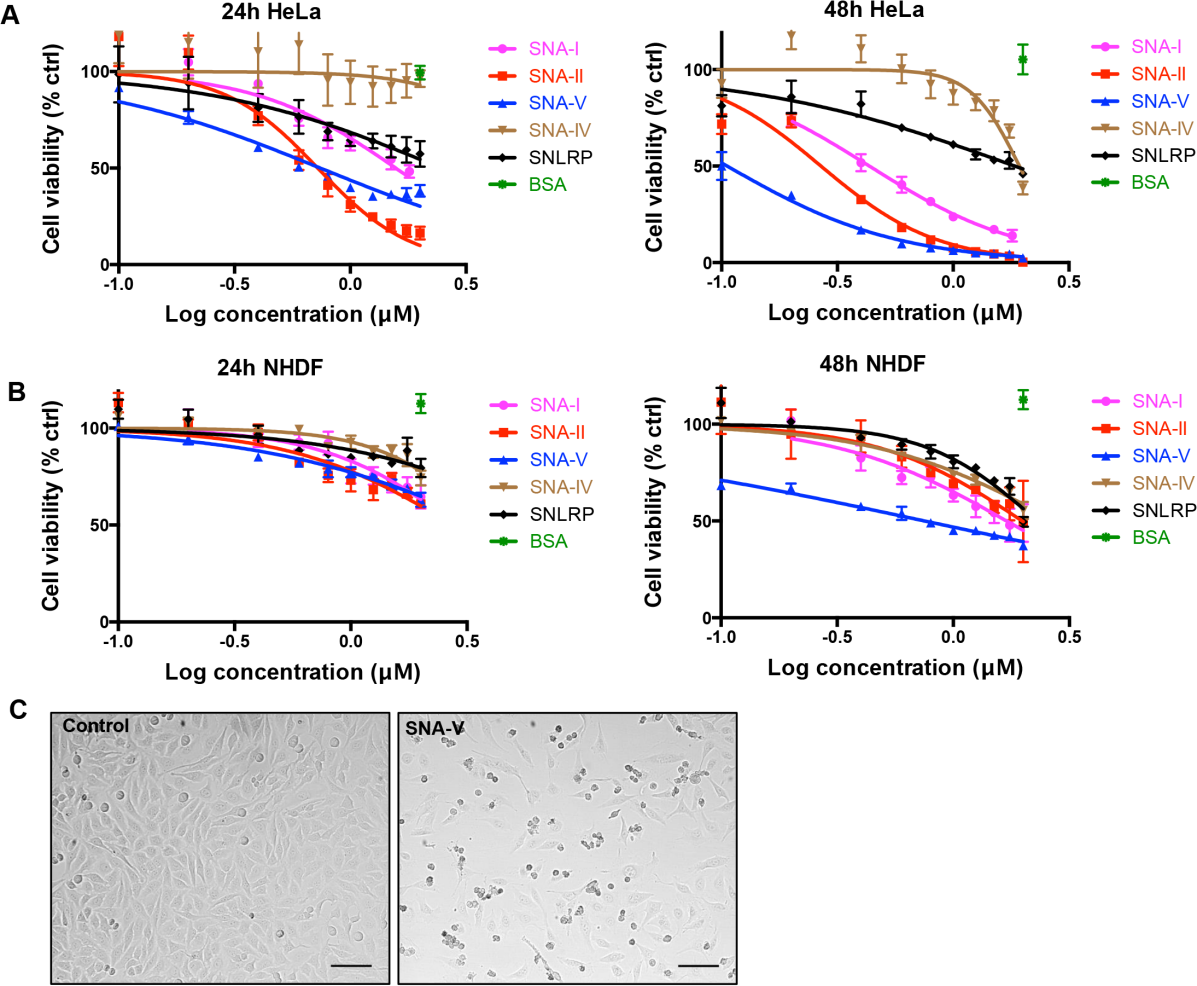
Dose response curve of the effect of *S*. *nigra* proteins on HeLa and NHDF cell viability after 24 and 48 h. (A) Dose-response curves for HeLa cells incubated for 24 and 48h with different concentrations of *S*. *nigra* RIPs/lectins. (B) Log concentration – cell viability curve of NHDF cells incubated for 24 and 48 h with different concentrations of *S*. *nigra* RIPs/lectins. % ctrl (treated/control X 100) = ratio of surviving treated cells/ surviving cells percent in control. All data are expressed as means ± SE of 3 biological replicates in 4 technical replicates (n = 12). (C) Transmission light microscopy images of HeLa cells grown in the absence (control) and presence of 1.5 μM SNA-V for 24 h. Scale bars represent 100 μm.

To assess the antiproliferative activity of the *S*. *nigra* RIPs (SNA-I, SNA-V and SNLRP) and lectins (SNA-II and SNA-IV) on HeLa and NHDF cells, spectrophotometric viability assays were performed after incubation with different concentrations (0.1–2 μM) of the proteins (Fig 2A and 2B). In HeLa cells, all proteins, except for SNA-IV, induced significant (p<0.05) cytotoxicity after 48 h incubation at the lowest protein concentration tested (0.1 μM). Furthermore, SNA-IV also became cytotoxic at concentrations > 1.5 μM. There was a clear dose- and time-dependent effect on HeLa cell viability. The degree of cytotoxicity after 48 h was as follows: SNA-V > SNA-II > SNA-I > SNLRP > SNA-IV (Table 1). The cytotoxic effect on HeLa cells was accompanied by clear morphological changes such as cell rounding and blebbing (Fig 2C). As evidenced by the increased LC50 values (Table 1), NHDF cells were much less susceptible to *S*. *nigra* proteins than HeLa cells. There was no statistically significant effect of SNA-IV and SNLRP on NHDF cell viability and proliferation after 24 h whereas all the other *S*. *nigra* proteins caused a significant cytotoxicity, though at much higher protein concentrations compared to HeLa cells. Only after 48 h, a significant effect on cell viability was witnessed for SNA-V (p<0.001) at the lowest protein concentration (0.05 μM).

**Table 1:** Comparison of LC50 values for the *S*. *nigra* proteins in HeLa and NHDF cell lines.

LC50 (μM)					
Time/Cell line	SNA-I	SNA-II	SNA-IV	SNA-V	SNLRP
**24h HeLa**	1.57 ± 0.32^c^	0.72 ± 0.11^a^	>2.00^b^	0.74 ± 0.08^a^	>2.00^b^
**48h HeLa**	0.43 ± 0.04^d^	0.27 ± 0.03^d^	1.932 ± 0.20^e^	0.11 ± 0.01^d^	1.85 ± 0.44^e^
**24h NHDF**	>2.00^f^	>2.00^f^	>2.00^f^	>2.00^f^	>2.00^f^
**48h NHDF**	1.70 ± 0.35^g, h^	2.00 ± 0.77^h^	>2.00^h^	0.74 ± 0.12^g^	>2.00^h^

Data are shown as means ± SE based on 4 replications per treatment, and each experiment was repeated 3 times. Different letters (a-h) represent significant cytotoxicity differences (Duncan; *P* < 0.05) between different *S*. *nigra* proteins under each treatment.

### *S*. *nigra* proteins are internalized by HeLa cells

**Fig 3:**
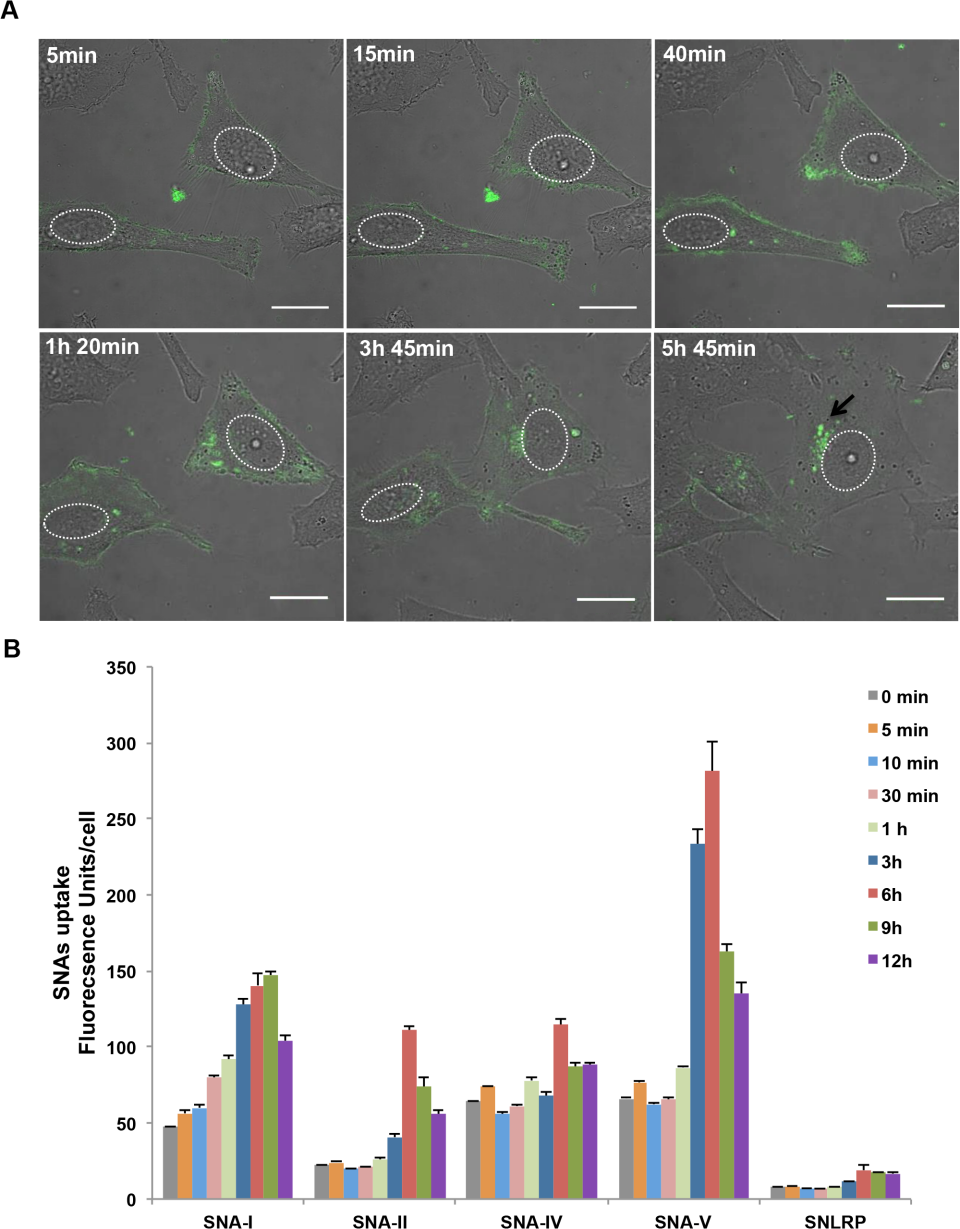
Internalization of FITC labeled *S*. *nigra* proteins in HeLa cells. (A) Confocal microscopic images of the uptake of SNA-I (25 nM) in HeLa cells after 6 hours incubation. The arrow indicates a spot where protein is accumulating. Nuclei have been delineated be in white. Scale bars represent 10 μm. (B) Uptake of 50 nM FITC labeled SNA-I, SNA-II, SNA-IV, SNA-V and SNLRP by HeLa cells after 0 min, 5 min, 30 min, 1 h, 3 h, 6 h, 9 h and 12 h incubation, based on fluorescence intensity, which was normalized by the FITC labeling efficiency. The pictures for quantification were acquired using identical confocal settings. Data are given as mean ± SE, based on at least 80 individual cell measurements per sample and each treatment was carried out with three independent replicates.

To find out whether differences in cytotoxicity between *S*. *nigra* proteins were due to differences in cellular protein uptake, HeLa cells were incubated with FITC labeled RIPs and lectins, and monitored using live cell confocal imaging (Fig 3A and S2 Fig). The binding and internalization kinetics of the proteins was quantified at different time points after incubation by measuring the intracellular fluorescence intensities (Fig 3B). Within 0–5 min, we measured fluorescent signals that co-aligned with the plasma membrane, presumably reflecting lectin binding. At later time points, signals were also observed at intracellular locations. All proteins showed time-dependent internalization kinetics with a maximum fluorescent signal after 6 to 9 h of incubation. When fluorescently labelled SNA-I was added to the medium of HeLa cells, the protein attached to the cell surface within minutes. Within the first 40 min, fluorescent SNA-I became internalized and increasingly accumulated in spots close to the nucleus up till 9h (Fig 3A). After 12h a decrease in fluorescent signal was observed, which probably reflects degradation and externalization of internalized lectin. A similar pattern was observed for SNA-V, albeit with a quicker turnover rate (max. at 6h) (S2B Fig). The perinuclear accumulation was maintained even after mitosis. SNA-II (S2A Fig) did not show a distinct tethering to the cell surface but also gradually accumulated inside the cell. Compared to SNA-V, intracellular accumulation of SNA-II was less pronounced. The same holds true for SNA-IV and SNLRP (S2C and S2D Fig, captured with much higher laser power setting to be visible): the amount of bound protein to the HeLa cell surface was almost undetectable and the amount of internalized protein was low compared to SNA-I (Fig 3B). After normalization for labeling efficiency, especially SNLRP internalization was negligible. During the time period of microscopic acquisition, HeLa cells incubated with the *S*. *nigra* proteins showed normal growth and cell division (S2B Fig). Only a small subset of cells incubated with SNA-I, SNA-II or SNA-V showed morphological changes characteristic for apoptosis (S2A Fig).

### *S*. *nigra* proteins predominantly localize to lysosomes but are also found in other organelles

**Fig 4:**
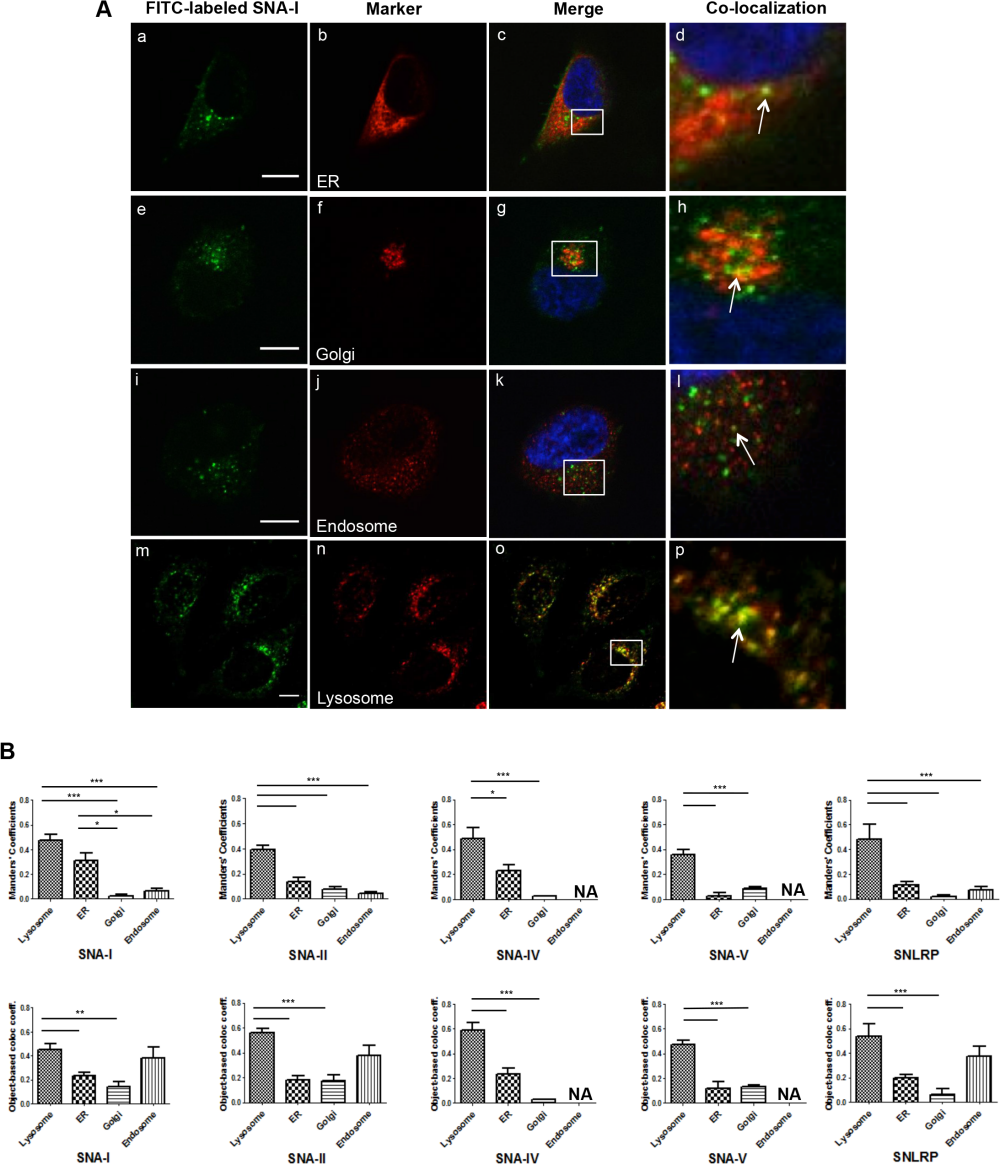
Confocal microscopic images and quantitative analysis of the colocalization. (A) Double immunofluorescence analysis of FITC-labeled SNA-I (a, e, i and m), and marker for ER (b, c and d), Golgi (f, g and h), endosomes (j, k and l) and lysosomes (n, o and p) in HeLa cells. The merged reconstructed images are shown in (d, h, l and p) with the green dots from FITC-labeled SNA-I and the red dots from the marker. The arrow indicates SNA-I dots overlapping with the marker. Scale bars represent 10 μm. (B) Manders’ coefficients and object-based colocalization graphs of the colocalization image analysis study. Asterisks denote values significantly different from the lysosome (*: p < 0,05; **: p< 0,01; ***: p < 0,001).

To find out where the FITC-labeled *S*. *nigra* proteins are targeted to in the cell, a quantitative colocalization analysis was performed on confocal microscopy images after co-labeling various endovesicles (Fig 4). Irrespective of the measurement method (Manders’ coefficients or object-based colocalized coefficients), the analysis showed that a few discrete dots overlap with markers for ER or Golgi but the majority of the lectin/RIP-positive dots colocalized with the endosomes and lysosomes. In the case of SNA-I and SNA-IV higher Manders’ coefficients were obtained for the ER, indicating relatively more colocalisation. It is also worth noting that the presence of SNA-IV and SNLRP in the Golgi compartment was clearly lower than for the other proteins.

### Autophagy induced in HeLa cells

**Fig 5:**
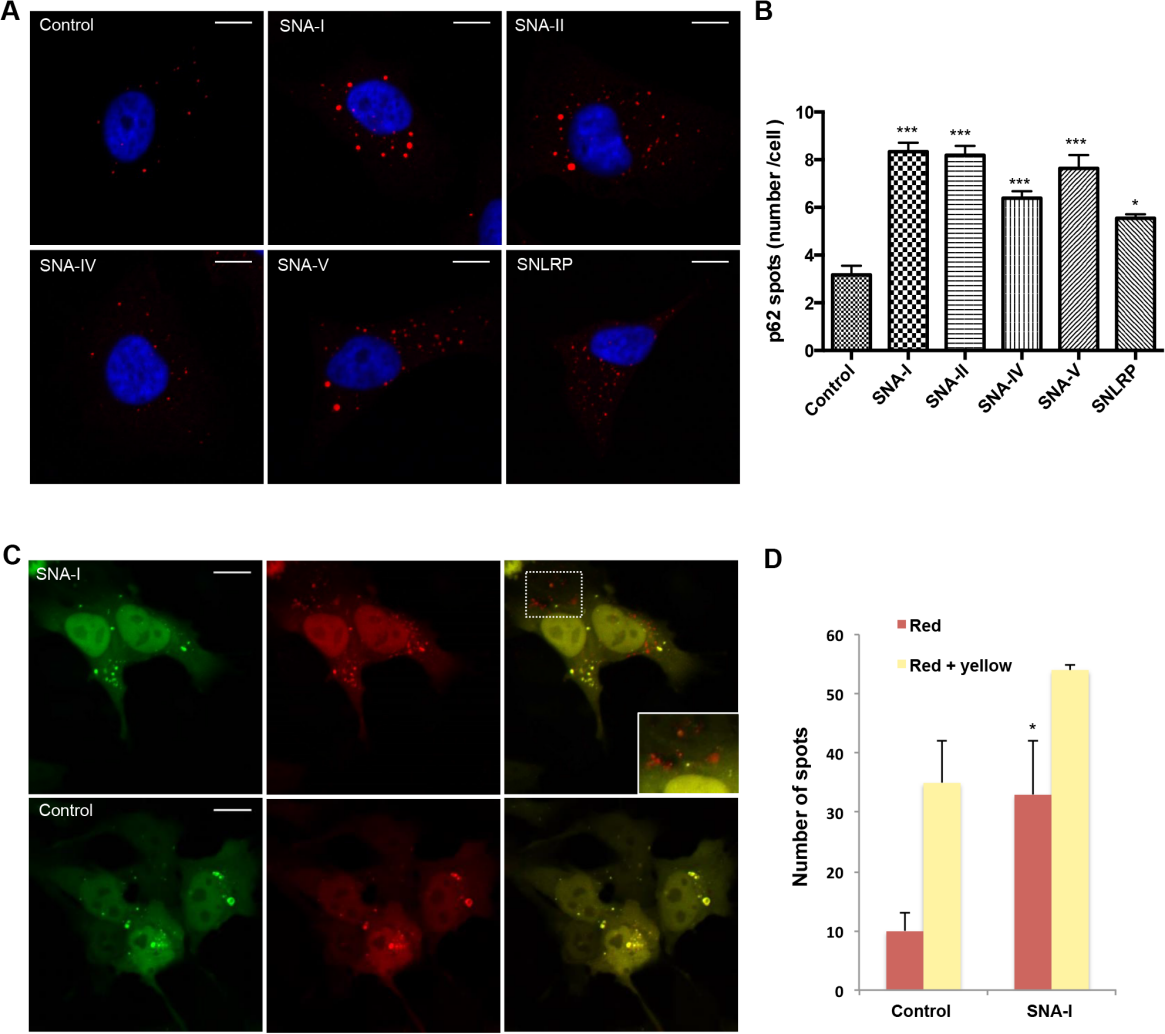
*S*. *nigra* proteins induce autophagy. (A) Confocal images of HeLa cells treated with *S*. *nigra* proteins, immuno-stained for p62 (red) and counterstained with DAPI (blue). (B) Quantification of p62 puncta numbers/cell (n>600 cells). Asterisks denote values significantly different from the cells incubated in the control (medium containing 1 x PBS) (*: p < 0,05; **: p< 0,01; ***: p < 0,001). (C) representative images of HeLa cells transfected with a tandem fluorescent mRFP-GFP-LC3 treated with 1x PBS (control) or SNA-I, (D) Quantification of yellow (autophagosomes) and red (autophagolysosomes) puncta reveals increased autophagy (total number of spots) as well as autophagic flux (red spots) in SNA-I treated cells. The asterisk indicates a significant difference compared to the control with P-value < 0.05. Scale bars in panels A and C represent 10 μm and 15 μm, respectively.

Given the fairly high load of proteins in the lysosomes, we reasoned that cellular uptake of some *S*. *nigra* proteins may trigger alternative degradation pathways such as autophagy. p62 is a direct substrate for autophagy that becomes included in autophagosomes, which is why we used it to monitor formation of autophagosomes [45]. A quantitative analysis of the number of p62 puncta per cell revealed that the autophagosome formation increased significantly after administration of any of the different elderberry proteins (Fig 5A and 5B). To determine whether the autophagic flux was altered, a tandem mRFP-GFP-tagged LC3 construct [34,46] was used. This fusion construct relies on the properties of mRFP to withstand the acidic environment of the lysosomes and maintain its fluorescence, whereas GFP does not. A strong increase in red over yellow (green+red) foci suggested enrichment of LC3 positive autophagolysosomes and thus an increase in autophagic flux in HeLa cells incubated with SNA-I (Fig 5C and 5D).

### Uptake of *S*. *nigra* proteins leads to translation inhibition *in cellula*

**Fig 6:**
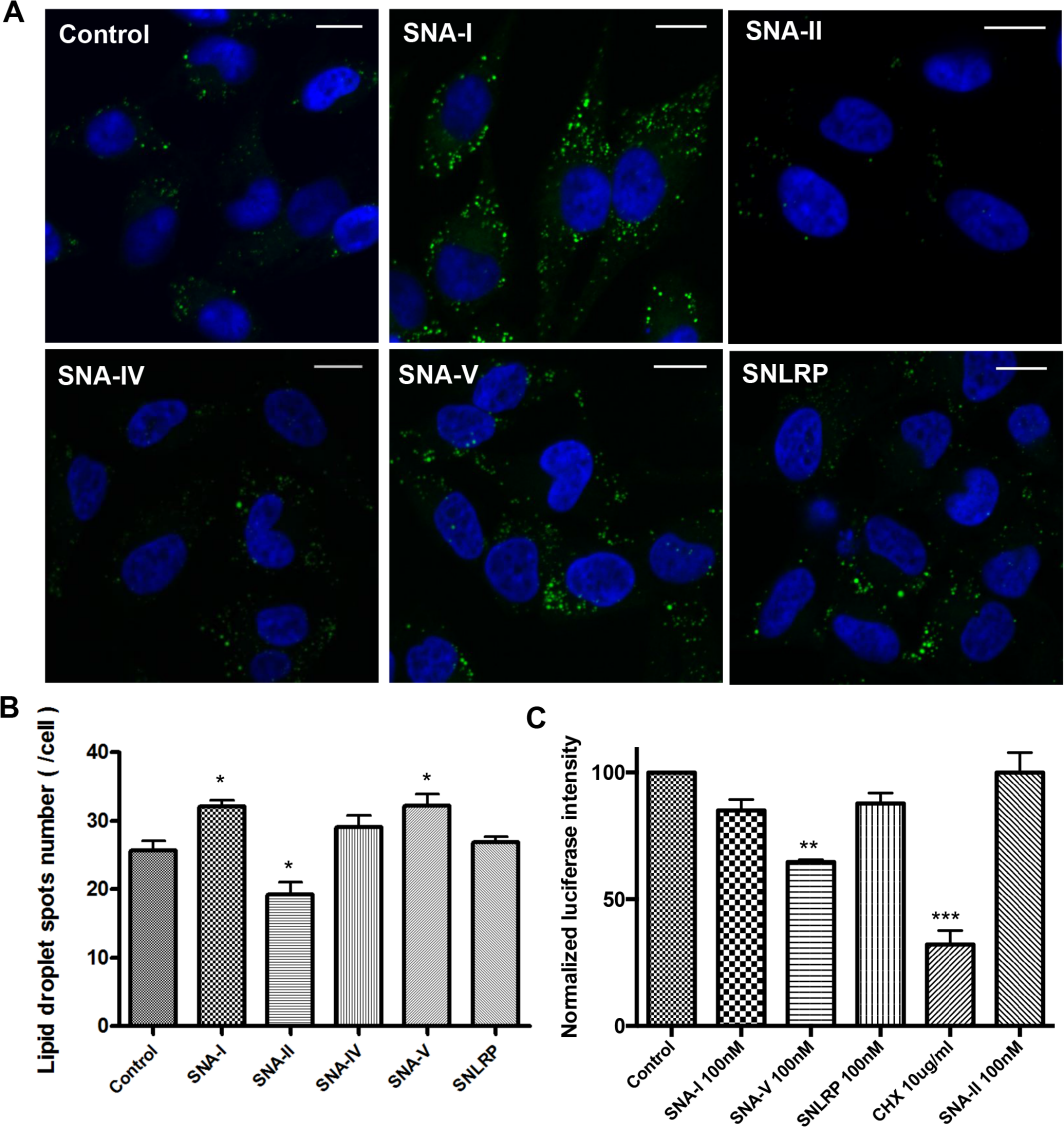
*In cellula* protein translation inhibition activity of *S*. *nigra* RIPs (A) Merged confocal images of HeLa cells stained with DAPI (blue) and BODIPY (green). Scale bars represent 15 μm. (B) Average numbers of LDs/cell (n>400) (C) Luciferase activity measured in HG1-luc2-IRES-tCD cells incubated with *S*. *nigra* RIPs/lectins and controls. The treatment with 1 x PBS was selected as the negative control, and cycloheximide was used as a positive control. The luminescent signal was normalized by the fluorescence signal from the Presto blue assay to correct for variations in cell density. Asterisks denote values significantly different from the cells incubated with control (1 x PBS) (*: p < 0,05; **: p< 0,01; ***: p < 0,001).

To ascertain protein translation inhibition activity within cells, we used the formation of lipid droplets (LDs) as a proxy [47]. HeLa cells have few small LDs when cultured in normal culture medium [48], but show strong accumulation of LD’s upon treatment with translation inhibitors such as cycloheximide [47]. When treated with 100 nM *S*. *nigra* RIPs SNA-I and SNA-V for 22 hours, there were significantly more LDs (Fig 6A and 6B). This was not the case for SNLRP and the non-RIP lectins (SNA-II and SNA-IV), suggesting that the *S*. *nigra* RIPs do effectively exert their ribosome inactivating activity inside the cells. Experiments with a bioluminescent reporter cell line (HG1-luc2-IRES-tCD) supported this notion [32] (Fig 6C). SNA-V significantly reduced the luminescent signal compared to the control. The other proteins showed no significant effect on the luminescent signal within the non-toxic range, possibly due to the weak sensitivity of the reporter cell line.

### Glycomic characterization of HeLa and NHDF cells

**Fig 7:**
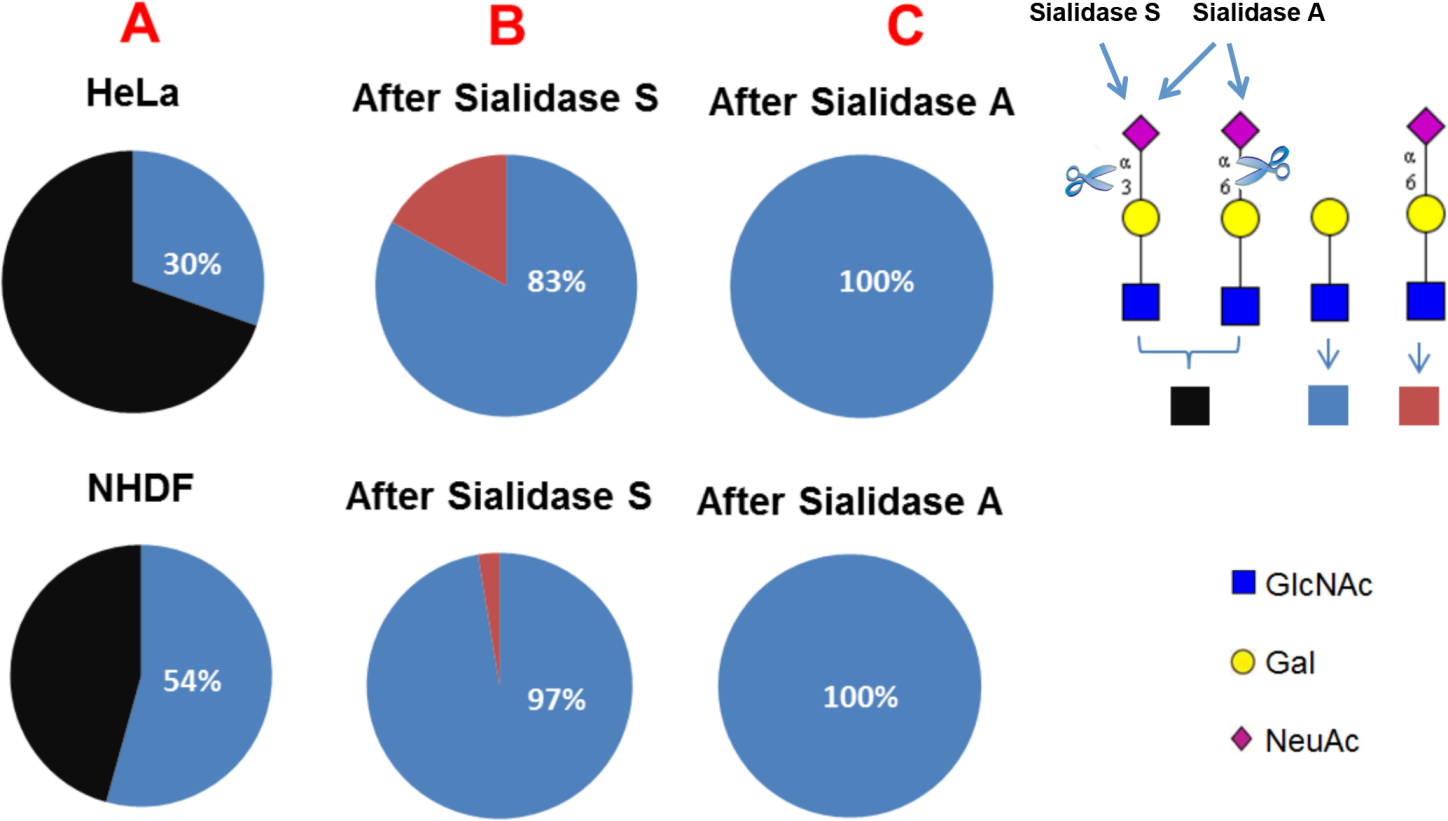
Comparative analysis of the relative intensities of LacNAc antenna and sialylated LacNAc antenna in all complex glycans in HeLa and NHDF cells (A), and in Sialidase S treated (B) and Sialidase A treated (C) samples. Black colour, relative intensity of α2–3 and α2–6 sialylated LacNAc antenna; blue colour, relative intensity of LacNAc antenna; red colour, relative intensity of α2–6 sialylated LacNAc antenna.

To assess whether differences in cytotoxicity between cell types and proteins were due to differences in carbohydrate binding to the cell surface, we analysed the glycome patterns on both glycoproteins and glycolipids of HeLa and NHDF cell samples using mass spectrometric methodologies [40]. These experiments confirmed the presence of high mannose and complex glycans in both cell types. The major complex N-glycans in both HeLa and NHDF cells are sialylated with N-acetylneuraminic acid (NeuAc) or are terminated with uncapped galactose (Gal) (S3 Fig). To determine the sialic acid linkages, the glycans were digested with sialidase S, which specifically removes α2–3 linked sialic acid, or sialidase A, which cleaves all non-reducing terminal sialic acid residues. Quantification of terminal Gal and terminal NeuAc on glycans from each cell line was performed by comparing the relative abundance of LacNAc (Gal-GlcNAc) antenna and sialylated LacNAc antenna. The result (Fig 7) showed that before Sialidase S digestion, the percentage of glycans terminated with Gal was approximately 54% in NHDF cells, which is significantly higher than in HeLa cells (approximately 30%). After Sialidase S digestion, the percentage of glycans terminated with α2–6 linked NeuAc in HeLa cells is around 17%, which is considerably higher than the 3% observed in NHDF cells, Sialidase A digestion removed all NeuAc supporting the glycomic assignments.

**Fig 8:**
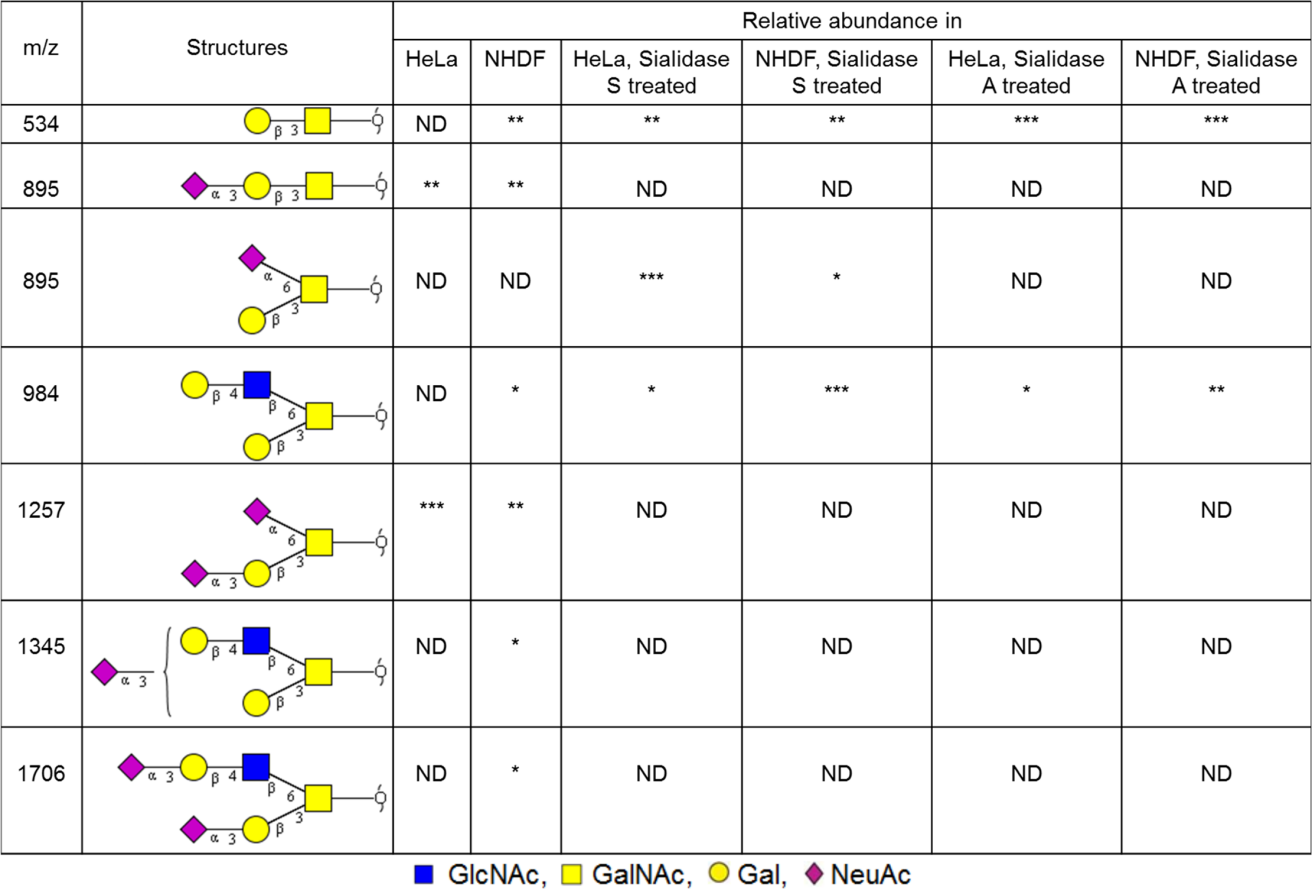
O-glycan structures observed in the MALDI-TOF MS spectra of Hela and NHDF glycoproteins. All glycans are permethylated and shown in the form of [M+Na]^+^. Glycan structures and linkages are drawn based on the molecular weight, O-glycan biosynthetic pathway and MS/MS data. ND, not detected. * = minor (<20%), ** = medium (20–50%), *** = major (>50%).

MALDI-TOF spectra of O-glycans from HeLa and NHDF cells are shown in S4 Fig The profile demonstrated the presence of both core 1 and core 2 structures in NHDF cells whilst in HeLa cells the majority of the glycan structures are of the core 1 type, although minor core 2 glycans were observed after sialidase digestion (Fig 8). In both cell types the O-glycans are either sialylated with NeuAc or terminated with uncapped Gal. Comparison of the abundances of the O-glycans (Fig 8) indicated that the glycans terminated with Gal are more prominent in NHDF cells than in HeLa cells, while the relative abundance of the glycans terminated with the α2–6 linked NeuAc (NeuAc(α2–6)GalNAc) is higher in HeLa cells.

**Fig 9:**
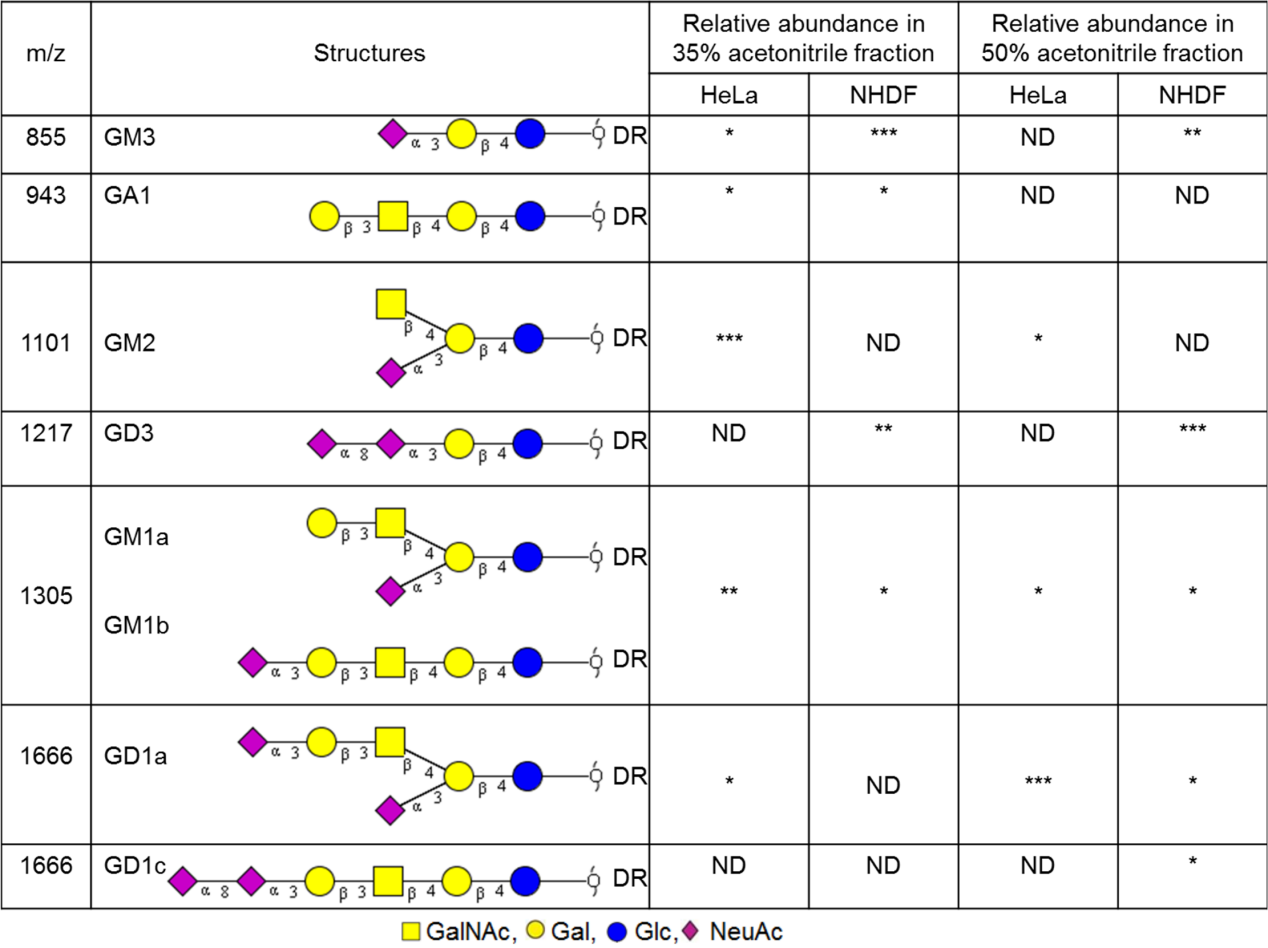
Structures of glycans derived from glycolipids observed in the MALDI-TOF MS spectra of Hela and NHDF cells. All glycans are deuteroreduced (DR), permethylated and shown in the form of [M+Na]^+^. These glycan structures and linkages are drawn based on the molecular weight, glycolipid glycan biosynthetic pathway and MS/MS data. ND, not detected. * = minor (<20%), ** = medium (20–50%), *** = major (>50%).

MALDI-TOF data from glycans derived from the glycolipids of HeLa and NHDF cells are shown in S5 Fig. Fig 9 summarises our structural conclusions which are derived from the glycomics experiments, taking into account biosynthetic considerations. The major glycans are sialylated, and glycans terminated with uncapped Gal represent only a minor fraction. In addition, a glycan (m/z 1101) terminated with HexNAc is present in HeLa cells. Sialidase digestion of HeLa samples confirmed the α2–3 linkage of the peripheral NeuAc of GM3, GM1b and GD1a (S6 Fig).

## Discussion

We have performed a comprehensive analysis of the cytotoxic activity of *S*. *nigra* proteins, including the type 2 RIPs (SNA-I, SNA-V and SNLRP) and lectins (SNA-II and SNA-IV), towards HeLa and NHDF cells. The results illustrate striking differences in terms of cytotoxicity and uptake between cell types and proteins. An important observation is that HeLa cell cultures are more sensitive to incubation with elderberry proteins than human fibroblasts. This can be explained by cell-type dependent differences at three levels, namely at the level of attachment, uptake and/or intracellular destination. Lectin binding to cell-type specific glycosylated proteins on the cell surface might lead to blocking of specific adhesion complexes or activate cell death factor receptors. For instance, galectin-1 induces apoptosis of activated T-cells and T leukaemia cell lines but does not affect resting T cells [49]. This difference in cytotoxicity depends on the expression of primary receptors (e.g. glycoproteins-CD43, CD45 and CD7) on the activated T-cell surface [50]. Specific (glycan-dependent) or aspecific (cell-type dependent) differences in endocytotic flux may in turn affect uptake efficiency and different cells may also sort or allocate the ingested proteins towards different organelles. Around 2000 genes are expressed at much higher levels in HeLa cells than in 16 normal human tissues (of which 805 are protein-coding). These genes typically relate to proliferation (cell cycle phase), transcription (RNA processing, rRNA transcription) and DNA repair [51]. Therefore, the specific cytotoxicity towards HeLa cells, may also point to a specific, modulatory role in cell proliferation and/or cell cycle-dependent processes. This notion also implies potential anti-carcinogenic activity of these elderberry proteins, which would enable the exploration of natural lectins as anti-cancer compounds.

The protein translation inhibition activity of SNLRP and SNA-V was significantly higher than that of SNA-I in the *in vitro* cell-free system. The IC50 values for SNA-I and SNA-V correspond well with earlier estimations derived from studies using rabbit reticulocyte lysates [11]. Barbieri et al. reported that the translation inhibition activity in a cell-free system was considerably higher for the reduced protein compared to the non-reduced type 2 RIPs (e.g. riproximin, ricin and volkensin) [10]. Similarly Voss et al. [20] reported a 2-fold higher activity for the purified A chain of the type 2 RIP from *Ximenia americana*, suggesting that the translation inhibition activity of the A-chain from this type 2 RIP is inhibited due to steric hindrance by the B-chain [20]. However, as also shown by Barbieri et al. [10] this result cannot readily be generalized since the reduction of type 2 RIPs such as SNA-I and SNLRP does not affect their activity on protein synthesis in a rabbit reticulocyte lysate. Our analyses revealed no significant influence of reduction on the activity of SNA-I, SNA-V and SNLRP, suggesting that there is no adverse impact of the lectin domain on the activity of these RIPs. Surprisingly, SNA-II also showed some modest RIP activity when tested at high concentration (>10 nM). This may be due to SNA-II binding to ribosomal proteins leading to inactivation of the ribosomes, but at present the exact modus operandi remains speculative.

Another surprising finding is that the order of the *in vitro* protein synthesis inhibition activity of *S*. *nigra* proteins (SNLRP > SNA-V > SNA-I > SNA-II > SNA-IV) did not mirror their cytotoxicity towards HeLa cells (SNA-V > SNA-II > SNA-I > SNLRP > SNA-IV). In line with this, it was shown that SNLRP exhibits at least 250-fold lower RIP activity *in cellula* than *in vitro* [52], while for ricin, RIP activity is in a comparable range *in cellula* and *in vitro* [11]. The lectin SNA-II proved to be even more cytotoxic than some of the RIPs, despite the absence of a RIP domain. This suggests that alternative or even complementary activities take place at the level of the cell. Several non-exclusive mechanisms that rely on lectin-carbohydrate interactions may explain this conundrum. One possibility is the differential uptake efficiency of the proteins in the cell; another is the inefficient targeting of the ribosomes (RIPs) or aspecific binding to and blocking of the ribosomes (lectins).

When assessing internalization kinetics, we observed that SNA-V is internalized most efficiently, whilst SNLRP was taken up least efficiently in HeLa cells. This could explain why these two proteins are at opposite ends of the cytotoxicity spectrum, despite their comparable *in vitro* protein translation inhibition activity. Indeed, lower binding to and uptake in the cells has been related to lower toxicity of type 2 RIPs, (e.g. ebulin 1) [53,54]. During cellular uptake, SNA-I, SNA-IV and SNA-V first bind to the cell surface and then accumulate around the nucleus. But, SNA-II and SNLRP enter the cells with much less attachment to the cell membrane. It is known that internalization of proteins in cells is not a must to provoke toxicity: some lectins can block cell surface receptors and thereby cause cytotoxicity [55]. In this respect, we note that SNA-V and SNA-II are Gal binding proteins whereas SNA-I and SNA-IV are sialic acid binding proteins. In contrast to the other proteins, SNLRP specifically recognizes GlcNAc oligomers. Thus, the differential cytotoxicity of the elderberry proteins could (in part) be caused by differences in the efficiency of binding to the cell membrane and the different number of binding sites present on the cell surface. To get a better view on the available glycan moieties, we performed a glycomic analyses. This revealed similar sialylation of the glycoproteins in the two cell types, a higher level of sialylation of the HeLa O-glycome compared with NHDF, and a variety of differences in the N-glycomes. In particular, N-glycomic analysis revealed that there were substantially more α2–6 linked sialic residues in glycoproteins from HeLa cells compared to NHDF cells. At least for SNA-I this could explain the increased cytotoxicity towards HeLa cells. Similarly, the higher overall level of sialylation of N- and O-glycans in HeLa cells compared with NHDF cells, could explain the higher cytotoxicity of SNA-IV for HeLa cells. SNA-II and SNA-V showed more cytotoxicity to the HeLa than to NHDF cell line, possibly evidenced by their specific binding to Core 1 glycans on glycolipids. Furthermore, SNA-V is the only protein that demonstrated significant cytotoxicity towards NHDF cells. This could be due to the high abundance of terminal Gal residues in these cells. SNLRP recognizes the core chitobiose moiety of N-glycan structures [21], so in theory, it could interact with all N-glycans. However, the LC50 values for SNLRP are very high, suggesting low binding to the cell surface glycoconjugates. Furthermore the amount of SNLRP internalized in HeLa cells was extremely low, suggesting that SNLRP binding to the chitobiose core is prevented e.g. by steric hindrance.

Although the MS data provided new insights on potential carbohydrate binding sites that correlate with cell binding/uptake for *S*. *nigra* proteins, it is clear that this interaction is complex and not sufficient to explain the cytotoxic activity of the proteins under study. For example, it remains unclear why the SNA-V and SNA-II are more toxic than SNA-I, despite the amount of terminal Gal on HeLa and NHDF cells being considerably lower than the amount of sialic acid.

When looking at the cellular destination via colocalization analysis, it became clear that most of the *S*. *nigra* proteins end up in the lysosomes. However, a small fraction, which varied per protein, also reached the Golgi apparatus or the ER. For ricin, the Golgi complex has typically been considered to be the crucial intermediate subcellular compartment for delivery into the cytosol [56], defining the cytotoxic potential of ricin [7,9,23]. Our results show low accumulation of the *S*. *nigra* proteins in the Golgi complex, but still significant toxicity. We reasoned that the protein fraction accumulating in the lysosomes may overload these organelles, causing saturation and subsequent activation of alternative degradation pathways such as autophagy. By scoring p62 positive foci, as well as LC3 foci, we confirmed an upregulation of the autophagic flux in the presence of all *S*. *nigra* proteins, suggesting that indeed such a pathway becomes activated, which in turn may provoke cell death [57]. Autophagic cell death pathways have also recently been described for Concanavalin A and *Polygonatum cyrtonema* lectin [16,17,58] and may present a valuable venue for novel cancer therapeutic strategies.

In summary, the cellular uptake of *S*. *nigra* RIPs and lectins follows differential routing depending on their molecular structures, carbohydrate-binding properties and interaction with glycans present on the cell surface, which together determine the cytotoxic activity. Until now, it was believed that the lectin domain only facilitated internalization of the proteins, but we now show that the lectin domain can also exert a cytotoxic activity as such. This inherent cytotoxic potential may be due to one or a combination of the following mechanisms: blocking cell membrane receptors, a synergistic role in protein translation inhibition activity and/or lysosome saturation triggering autophagy. Our data helps filling in pieces of the puzzle of RIP induced cell death. Furthermore, a better understanding of the interaction of RIPs with a variety of carbohydrate-binding properties with different cell types may contribute to the development of more specific immunotoxins for future medical applications.

## Supporting Information

S1 Fig SDS-Polyacrylamide gel electrophoresis of *S*. *nigra* proteins. (A) Purified *S*. *nigra* proteins were analyzed under non-reducing (left) and reducing conditions (with 2% β-mercaptoethanol) (right). Samples (3 μg) were loaded as follows: lane 1- Page Ruler Prestained Protein Ladder (Fermentas); lane 2- SNA-I; lane 3- SNA-V; lane 4- SNLRP; lane 5-SNA-II; lane 6- SNA-IV. (B) Non-reduced (without treatment) and reduced (incubation with 0,025 M DTT at 37°C for 1 h or 2 h) *S*. *nigra* proteins for *in vitro* protein synthesis inhibition assay. Samples (7.5 μg) were loaded as follows: Lane 1- Page Ruler Prestained Protein Ladder; lane 2, 5 and 8- non-reduced SNA-I, SNA-V and SNLRP, respectively; lane 3, 6 and 9- reduced SNA-I, SNA-V and SNLRP treated with DTT for 1 h 37°C, respectively; lane 4, 7 and 10- reduced SNA-I, SNA-V and SNLRP treated with DTT for 2 h at 37°C, respectively.
(TIF)

S2 Fig Internalization of FITC labelled *S*. *nigra* proteins in HeLa cells at different time points during a long incubation period. Confocal microscopic images of the uptake of SNA-II (A), SNA-V (B), SNLRP (C) and SNA-IV (D) in HeLa cells during incubation for a maximum of 8 hours. A small subset of HeLa cells incubated with SNA-II and SNA-V showed morphological changes characteristic for mitosis and apoptosis, which were showed by arrows. To visualize the cellular uptake clearly, the live cell images were captured with different settings (much higher laser power settings for SNA-IV and SNLRP and gain visibility). Scale bars represent 20μm.
(TIF)

S3 Fig Annotated MALDI-TOF MS spectra of permethylated N-glycans in Hela (A) and NHDF (B) cells. Profiles were obtained from the 50% acetonitrile fraction from a C18 Sep-Pak column. All ions are [M+Na]^+^. Putative structures are based on the molecular weight, N-glycan biosynthetic pathway and MS/MS data.
(TIF)

S4 Fig Annotated MALDI-TOF MS spectra of permethylated O-glycans in Hela (A) and NHDF (B) cells. Profiles were obtained from the 35% acetonitrile fraction from a C18 Sep-Pak column. All ions are [M+Na]^+^. Putative structures are based on the molecular weight, O-glycan biosynthetic pathway and MS/MS data.
(TIF)

S5 Fig Annotated MALDI-TOF MS spectra of deuteroreduced, permethylated glycolipid derived glycans from Hela (A, B) and NHDF (C, D) cells. These profiles were obtained from the 35% and 50% acetonitrile fractions from a C18 Sep-Pak column. All ions are [M+Na]^+^. Putative structures are based on the molecular weight, glycolipid glycan biosynthetic pathway and MS/MS data.
(TIF)

S6 Fig Structures of glycans derived from glycolipids observed in the MALDI-TOF MS spectra of HeLa cells. All glycans are deuteroreduced (DR), permethylated and [M+Na]^+^. Glycan structures are drawn based on molecular weight, glycolipid glycan biosynthetic pathway and MS/MS data. ND, not detected. * = minor (<20%), ** = medium (20–50%), *** = major (>50%).
(TIF)

## References

[1] Peumans WJ, Hao Q, Van Damme EJM. Ribosome-inactivating proteins from plants: more than RNA *N*-glycosidases? FASEB J. 2001;15:1493–1506. 11427481 10.1096/fj.00-0751rev

[2] Stirpe F, Battelli MG. Ribosome-inactivating proteins: progress and problems. Cell Mol Life Sci. 2006;63:1850–1866. 16799768 10.1007/s00018-006-6078-7 PMC11136412

[3] Van Damme EJM, Hao Q, Barre A, Vandenbussche F, Desmyter S, Rougé P, et al Ribosome-inactivating proteins: a family of plant proteins that do more than inactivate ribosomes. Crit Rev Plant Sci. 2001;20:395–465.

[4] Puri M, Kaur I, Perugini MA, Gupta RC. Ribosome-inactivating proteins: current status and biomedical applications. Drug Discov. Today 2012;17:774–83. 10.1016/j.drudis.2012.03.007 22484096

[5] Ferreras JM, Citores L, Iglesias R, Jiménez P, Girbés T. Use of ribosome-inactivating proteins from *Sambucus* for the construction of immunotoxins and conjugates for cancer therapy. Toxins 2011;3:420–41. 10.3390/toxins3050420 22069717 PMC3202832

[6] Sandvig K, Olsnes S, Pihl A. Binding, uptake and degradation of the toxic proteins abrin and ricin by toxin-resistant cell variants. Eur J Biochem. 1978;82:13–23. 620666 10.1111/j.1432-1033.1978.tb11992.x

[7] Sandvig K, Prydz K, Hansen SH, Van Deurs B. Ricin transport in brefeldin A-treated cells: correlation between Golgi structure and toxic effect. J Cell Biol. 1991;115:971–981. 1955466 10.1083/jcb.115.4.971 PMC2289950

[8] Lord JM, Roberts LNM. Toxin entry: retrograde transport through the secretory pathway. J Cell Biol. 1998;140:733–736. 9472027 10.1083/jcb.140.4.733 PMC2141750

[9] Yoshida T, Chen CC, Zhang MS, Wu HC. Disruption of the Golgi apparatus by brefeldin A inhibits the cytotoxicity of ricin, modeccin, and *Pseudomonas* toxin. Exp Cell Res. 1991;192:389–395. 1899070 10.1016/0014-4827(91)90056-z

[10] Barbieri L, Ciani M, Girbés T, Liu W, Van Damme EJM, Peumans WJ. Enzymatic activity of toxic and non-toxic type 2 ribosome-inactivating proteins. FEBS Lett. 2004;563:219–222. 15063752 10.1016/S0014-5793(04)00286-8

[11] Jiménez P, Gayoso MJ, Girbés T. Non-toxic type 2 ribosome-inactivating proteins In: Stirpe F, Lappi DA, editors. Ribosome-inactivating proteins: ricin and related proteins. New Jersey: Wiley Blackwell Press; 2014 p. 67–82.

[12] Battelli MG, Citores L, Buonamici L, Ferreras JM, de Benito FM, Stirpe F, et al Toxicity and cytotoxicity of nigrin b, a two-chain ribosome-inactivating protein from *Sambucus nigra*: a comparison with ricin. Arch. Toxicol. 1997;71:360–364. 9195017 10.1007/s002040050399

[13] Das MK, Sharma RS, Mishra V. Induction of apoptosis by ribosome inactivating proteins: Importance of N-glycosidase activity. Appl Biochem Biotechnol. 2012;166:1552–1561. 10.1007/s12010-012-9550-x 22262020

[14] Narayanan S, Surendranath K, Bora N, Surolia A, Anjali AK. Ribosome inactivating proteins and apoptosis. FEBS Lett. 2005;579:1324–1331. 15733836 10.1016/j.febslet.2005.01.038

[15] Sikriwal D, Batra JK. Ribosome inactivating proteins and apoptosis In: Lord JM, Hartley MR, editors. Plant cell monographs. Toxic plant proteins. Berlin: Springer-Verlag; 2010 p. 107–132.

[16] Fu LL, Zhou CC, Yao S, Yu JY, Liu B, Bao JK. Plant lectins: targeting programmed cell death pathways as antitumor agents. Int. J. Biochem. Cell Biol. 2011;43:1442–1449. 10.1016/j.biocel.2011.07.004 21798364

[17] Liu B, Bian HJ, Bao JK. Plant lectins: potential antineoplastic drugs from bench to clinic. Cancer Lett. 2010;287:1–12. 10.1016/j.canlet.2009.05.013 19487073

[18] Fang EF, Zhang CZY, Ng TB, Wong JH, Pan WL, Ye XJ. *Momordica Charantia* lectin, a type II ribosome inactivating protein, exhibits antitumor activity toward human nasopharyngeal carcinoma cells *in vitro* and *in vivo* . Cancer Prev Res. 2012;5:109–121. 10.1158/1940-6207.CAPR-11-0203 21933914

[19] Gadadhar S, Karande AA. Abrin immunotoxin: targeted cytotoxicity and intracellular trafficking pathway. PloS One, 2013;8:e58304–e58319. 10.1371/journal.pone.0058304 23472175 PMC3589266

[20] Voss C, Eyol E, Frank M, Von Der Lieth CW, Berger MR. Identification and characterization of riproximin, a new type II ribosome-inactivating protein with antineoplastic activity from *Ximenia americana* . FASEB J. 2006;20:1194–1196. 16641197 10.1096/fj.05-5231fje

[21] Shang C, Van Damme EJM. Comparative analysis of carbohydrate binding properties of *Sambucus nigra* lectins and ribosome-inactivating proteins. Glycoconj J. 2014;31:345–354. 10.1007/s10719-014-9527-9 24853865

[22] Battelli MG, Musiani S, Buonamici L, Santi S, Riccio M, Maraldi NM, et al Interaction of volkensin with HeLa cells: binding, uptake, intracellular localization, degradation and exocytosis. Cell Mol Life Sci. 2004;61:1975–1984. 15289938 10.1007/s00018-004-4171-3 PMC11138743

[23] Citores L, Ferreras JM, Muñoz R, Benítez J, Jiménez P, Girbés T. Targeting cancer cells with transferrin conjugates containing the non-toxic type 2 ribosome-inactivating proteins nigrin b or ebulin 1. Cancer Lett. 2002;184:29–35. 12104045 10.1016/s0304-3835(02)00169-6

[24] Ferreras JM, Citores L, Iglesias R, Jiménez P, Girbés T. Use of ribosome-inactivating proteins from *Sambucus* for the construction of immunotoxins and conjugates for cancer therapy. Toxins 2011;3:420–41. 10.3390/toxins3050420 22069717 PMC3202832

[25] Tejero J, Jiménez P, Quinto EJ, Cordoba-Diaz D, Garrosa M, Cordoba-Diaz M, et al Elderberries: A source of ribosome-inactivating proteins with lectin activity. Molecules 2015;20:2364–2387. 10.3390/molecules20022364 25647575 PMC6272206

[26] Svinth M, Steighardt J, Hernandez R, Suh JK, Kelly C, Day P. Differences in cytotoxicity of native and engineered RIPs can be used to assess their ability to reach the cytoplasm. Biochem Biophys Res Commun. 1998;249:637–642. 9731188 10.1006/bbrc.1998.9207

[27] Broekaert WF, Nsimba-Lubaki M, Peeters B, Peumans WJ. A lectin from elder (*Sambucus nigra* L.) bark. Biochem J. 1984;221:163–169. 6466312 10.1042/bj2210163 PMC1144016

[28] Mach L, Scherf W, Ammann M, Poetsch J, Bertsch W, März L, Glössl J. Purification and partial characterization of a novel lectin from elder (*Sambucus nigra L*.) fruit. Biochem J. 1991;278:667–671. 1910334 10.1042/bj2780667 PMC1151398

[29] Van Damme EJM, Rougé P, Barre A, Van Leuven F, Peumans WJ. Characterization and molecular cloning of *Sambucus nigra* agglutinin V (nigrin b), a GalNAc-specific type-2 ribosome-inactivating protein from the bark of elderberry (*Sambucus nigra*). Eur J Biochem. 1996;237:505–513. 8647092 10.1111/j.1432-1033.1996.0505k.x

[30] Van Damme EJM, Rougé P, Barre A, Van Leuven F, Peumans WJ. Isolation and molecular cloning of a novel type 2 ribosome-inactivating protein with an inactive B chain from elderberry (*Sambucus nigra*) Bark. J Biol Chem. 1997;272:8353–8360. 9079659 10.1074/jbc.272.13.8353

[31] Emmanuel F, Turpin E, Alfsen A, Frénoy JP. Separation of ricin A- and B-chains after dithiothreitol reduction. Anal Biochem. 1988;173:134–141. 3189793 10.1016/0003-2697(88)90170-4

[32] Jiménez AZ, Gressette M, Barjon C, Wei M, Gourzones C, Busson P. Rapid obtention of stable, bioluminescent tumor cell lines using a tCD2-luciferase chimeric construct. BMC Biol. 2011;11:26–35. 10.1186/1472-6750-11-26 PMC3078863 21435248

[33] Oliveira C, Nicolau A, Teixeira JA, Domingues L. Cytotoxic effects of native and recombinant frutalin, a plant galactose-binding lectin, on HeLa cervical cancer cells. J Biomed Biotechnol. 2011;2011:568932–568941. 10.1155/2011/568932 22131813 PMC3206378

[34] Kimura S, Noda T, Yoshimori T. Dissection of the autophagosome maturation processby a novel reporter protein, tandem fluorescent-tagged LC3. Autophagy 2007;3:452–460. 17534139 10.4161/auto.4451

[35] Manders EMM, Verbeek FJ, Aten JA. Measurement of co-localization of objects in dual-colour confocal image. J Microsc. 1993;169:375–382. 10.1111/j.1365-2818.1993.tb03313.x 33930978

[36] Bolte S, Cordelières FP. A guided tour into subcellular colocalization analysis in light microscopy. J Microsc. 2006;224:213–232. 17210054 10.1111/j.1365-2818.2006.01706.x

[37] Verdoodt F, Willems M, Dhondt I, Houthoofd W, Bert W, De Vos WH. Measurement of S-phase duration of adult stem cells in the flatworm *Macrostomum lignano* by double replication labelling and quantitative colocalization analysis. Cell Biol Int. 2012;36:1251–1259. 10.1042/CBI20120187 23005924

[38] De Vos WH, Van Neste L, Dieriks B, Joss GH, Van Oostveldt P. High content image cytometry in the context of subnuclear organization. Cytometry Part A 2010;77A:64–75. 10.1002/cyto.a.20807 19821512

[39] Jang-Le J, North SJ, Sutton-Smith M, Goldberg D, Panico M, Morris H, et al Glycomic profiling of cells and tissues by mass spectrometry: fingerprinting and sequencing methodologies. Meth Enzymol. 2006;415:59–86. 17116468 10.1016/S0076-6879(06)15005-3

[40] North SJ, Jang-Lee J, Harrison R, Canis K, Ismail MN, Trollope A, et al Mass spectrometric analysis of mutant mice. Methods Enzymol. 2010;478:27–77. 10.1016/S0076-6879(10)78002-2 20816474

[41] Jia N, Barclay WS, Roberts K, Yen HL, Chan RW, Lam AK, et al Glycomic characterisation of respiratory tract tissues of ferrets: implications for its use in influenza virus infection studies. J Biol Chem. 2014;289:28489–28504. 10.1074/jbc.M114.588541 25135641 PMC4192499

[42] Ceroni A, Maass K, Geyer H, Geyer R, Dell A, Haslam SM. GlycoWorkbench: A tool for the computer-assisted annotation of mass spectra of glycans. J Proteome Res. 2008;7:1650–1659. 10.1021/pr7008252 18311910

[43] Schachter H. The 'yellow brick road' to branched complex N-glycans. Glycobiology 1991;1:453–461. 1840403 10.1093/glycob/1.5.453

[44] Taylor ME, Drickamer K. Introduction to glycobiology 3rd ed. United Kingdom: Oxford University Press; 2011 p. 24, 37, 54–55.

[45] Bjørkøy G, Lamark T, Brech A, Outzen H, Perander M, Øvervatn A, et al p62/SQSTM1 forms protein aggregates degraded by autophagy and has a protective effect on huntingtin-induced cell death. J Cell Biol. 2005;171:605–614. 10.1083/jcb.200507002 PMC2171557 16286508

[46] Mizushima N, Yoshimorim T, Levine B. Methods in mammalian autophagy research. Cell 2010;140:313–326. 10.1016/j.cell.2010.01.028 20144757 PMC2852113

[47] Suzuki H. Translation inhibitors induce formation of cholesterol ester-rich lipid droplets. PLoS One 2012;7:42379–42387. 10.1371/journal.pone.0042379 PMC3411751 22879956

[48] Shibata M, Yoshimura K, Tamura H, Ueno T, Nishimura T, Inoue T, et al LC3, a microtubule-associated protein1A/B light chain3, is involved in cytoplasmic lipid droplet formation. Biochem Biophys Res Commun. 2010;393:274–279. 10.1016/j.bbrc.2010.01.121 20132792

[49] Perillo NL, Pace KE, Seilhamer JJ, Baum LG. Apoptosis of T cells mediated by galectin-1. Nature 1995;378:736–739. 7501023 10.1038/378736a0

[50] Rabinovich GA. Galectin-1 as a potential cancer target. Br J Cancer 2005;92:1188–1192. 15785741 10.1038/sj.bjc.6602493 PMC2361964

[51] Landry JJ, Pyl PT, Rausch T, Zichner T, Tekkedil MM, Stütz AM, et al The genomic and transcriptomic landscape of a HeLa cell line. G3(Bethesda). 2013;3:1213–1224. 23550136 10.1534/g3.113.005777 PMC3737162

[52] Battelli MG, Barbieri L, Bolognesi A, Buonamici L, Valbonesi P, Polito L, et al Ribosome-inactivating lectins with polynucleotide: adenosine glycosidase activity. FEBS L. 1997;408:355–359. 10.1016/s0014-5793(97)00463-8 9188793

[53] Citores L, Munoz R, De Benito FM, Iglesias R, Ferreras JM, et al Differential sensitivity of HELA cells to the type 2 ribosome-inactivating proteins ebulin l, nigrin b and nigrin f as compared with ricin. Cell Mol Biol. (Noisy-le-Grand, France) 1996;42:473–476. 8828902

[54] Pascal JM, Day PJ, Monzingo AF, Ernst SR, Robertus JD, Iglesias R, et al 2.8-Å crystal structure of a nontoxic type-II ribosome-inactivating protein, ebulin l. Proteins: Structure, Function, and Bioinformatics 2001;43:319–326. 10.1002/prot.1043 11288182

[55] Lichtenstein RG, Rabinovich GA. Glycobiology of cell death: when glycans and lectins govern cell fate. Cell Death Differ. 2013;20:976–986. 10.1038/cdd.2013.50 23703323 PMC3705604

[56] Van Deurs B, Petersen OW, Olsnes S, Sandvig K. Delivery of internalized ricin from endosomes to cisternal Golgi elements is a discontinuous, temperature-sensitive process. Exp Cell Res. 1987;171:137–152. 3622628 10.1016/0014-4827(87)90257-6

[57] Klionsky DJ, Abdalla FC, Abeliovich H, Abraham RT, Acevedo-Arozena A, Adeli K, et al Guidelines for the use and interpretation of assays for monitoring autophagy. Autophagy 2012;8:445–544. 22966490 10.4161/auto.19496 PMC3404883

[58] Lei HY, Chang CP. Lectin of Concanavalin A as an anti-hepatoma therapeutic agent. J Biomed Sci. 2009;16:10–1186. 10.1186/1423-0127-16-10 19272170 PMC2644972

