# Supplementary figures and images for: The Cytotoxicity of Elderberry Ribosome-Inactivating Proteins Is Not Solely Determined by Their Protein Translation Inhibition Activity
Source: PLoS One. 2015 Jul 6;10(7):e0132389. doi: 10.1371/journal.pone.0132389 (PMC4493096; doi:10.1371/journal.pone.0132389)

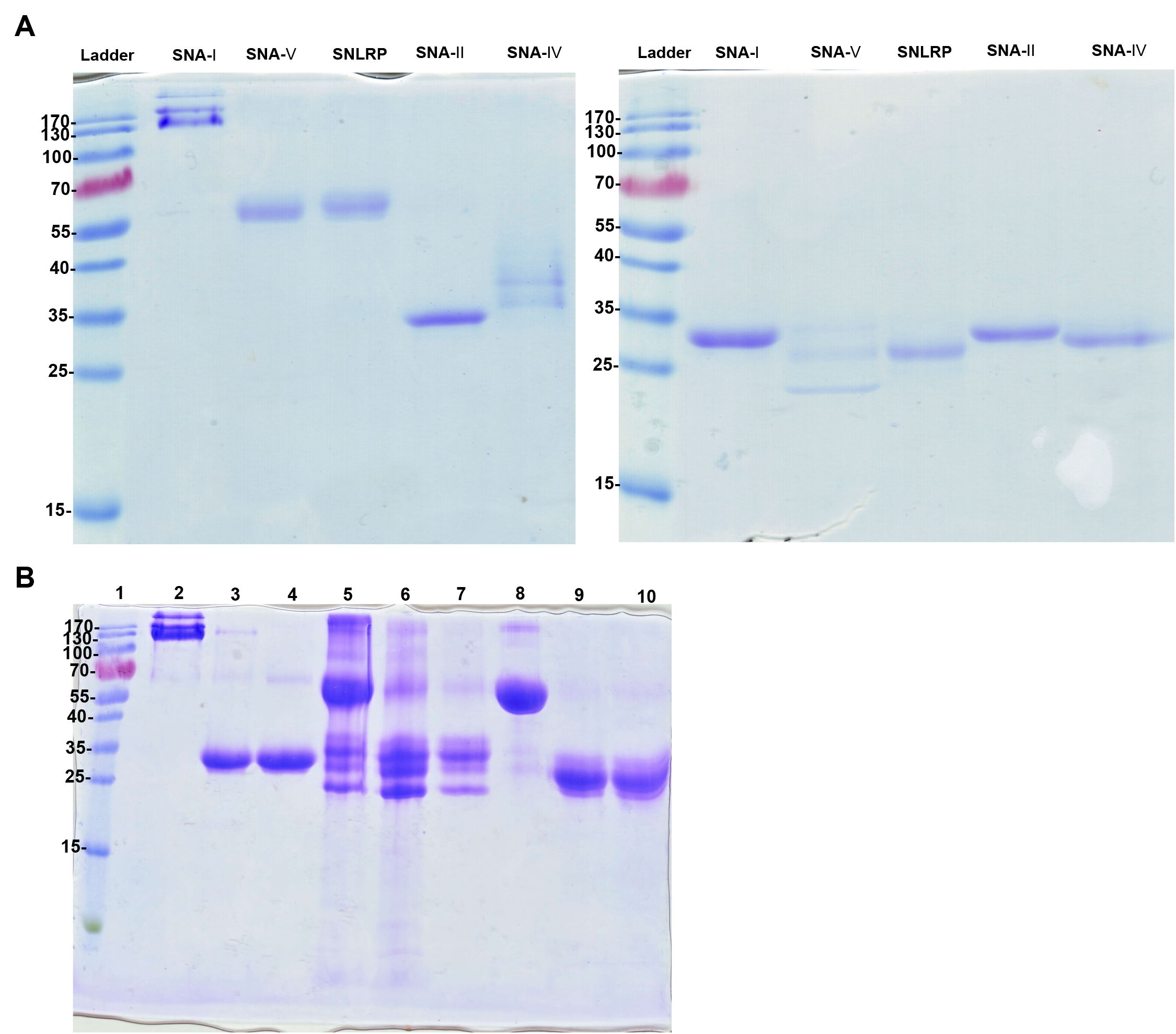

Supplement: S1 Fig — (A) Purified S. nigra proteins were analyzed under non-reducing (left) and reducing conditions (with 2% β-mercaptoethanol) (right). Samples (3 μg) were loaded as follows: lane 1- Page Ruler Prestained Protein Ladder (Fermentas); lane 2- SNA-I; lane 3- SNA-V; lane 4- SNLRP; lane 5-SNA-II; lane 6- SNA-IV. (B) Non-reduced (without treatment) and reduced (incubation with 0,025 M DTT at 37°C for 1 h or 2 h) S. nigra proteins for in vitro protein synthesis inhibition assay. Samples (7.5 μg) were loaded as follows: Lane 1- Page Ruler Prestained Protein Ladder; lane 2, 5 and 8- non-reduced SNA-I, SNA-V and SNLRP, respectively; lane 3, 6 and 9- reduced SNA-I, SNA-V and SNLRP treated with DTT for 1 h 37°C, respectively; lane 4, 7 and 10- reduced SNA-I, SNA-V and SNLRP treated with DTT for 2 h at 37°C, respectively. (TIF) [file pone.0132389.s001.tif]

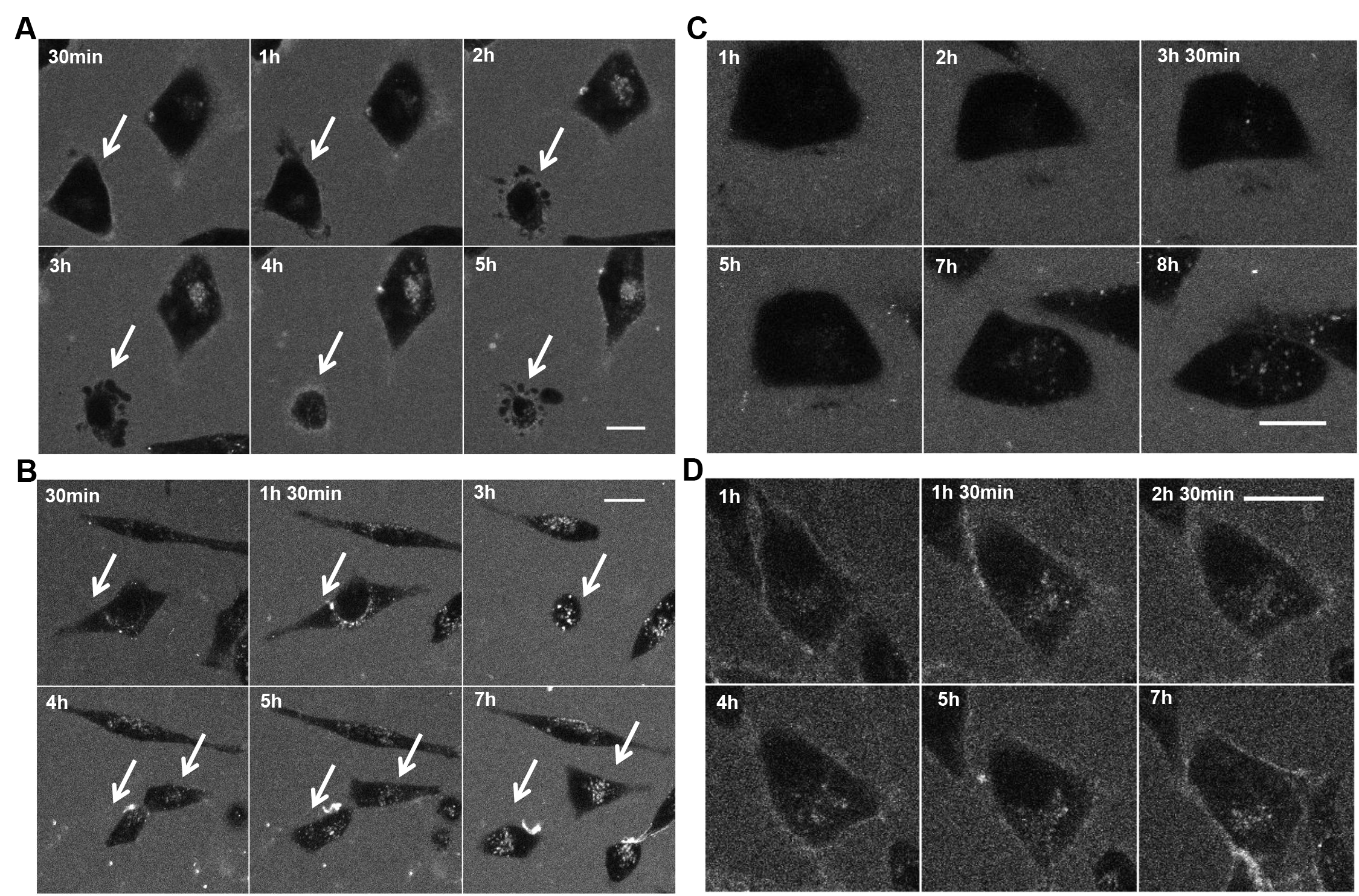

Supplement: S2 Fig — Confocal microscopic images of the uptake of SNA-II (A), SNA-V (B), SNLRP (C) and SNA-IV (D) in HeLa cells during incubation for a maximum of 8 hours. A small subset of HeLa cells incubated with SNA-II and SNA-V showed morphological changes characteristic for mitosis and apoptosis, which were showed by arrows. To visualize the cellular uptake clearly, the live cell images were captured with different settings (much higher laser power settings for SNA-IV and SNLRP and gain visibility). Scale bars represent 20μm. (TIF) [file pone.0132389.s002.tif]

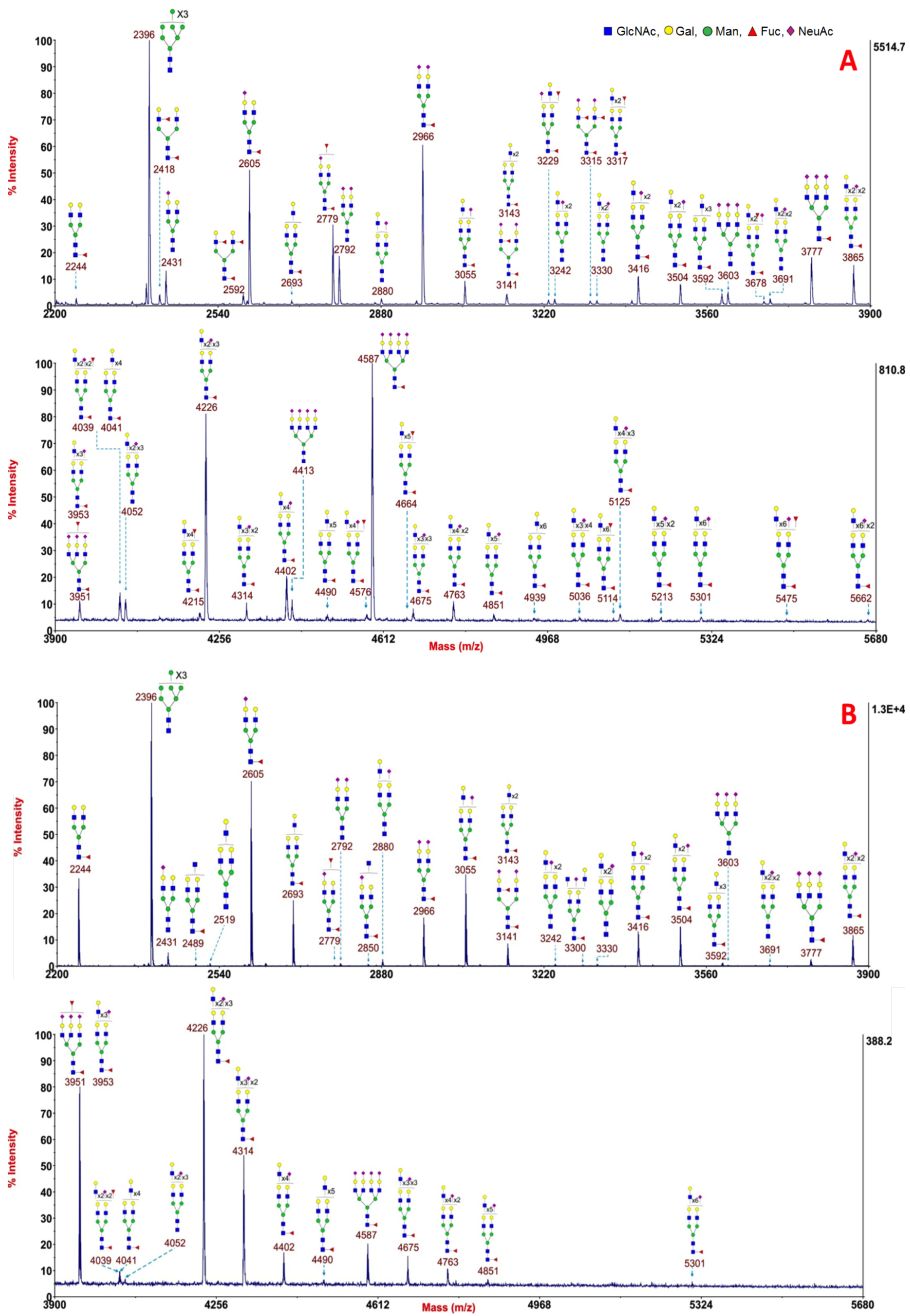

Supplement: S3 Fig — Profiles were obtained from the 50% acetonitrile fraction from a C18 Sep-Pak column. All ions are [M+Na]+. Putative structures are based on the molecular weight, N-glycan biosynthetic pathway and MS/MS data. (TIF) [file pone.0132389.s003.tif]

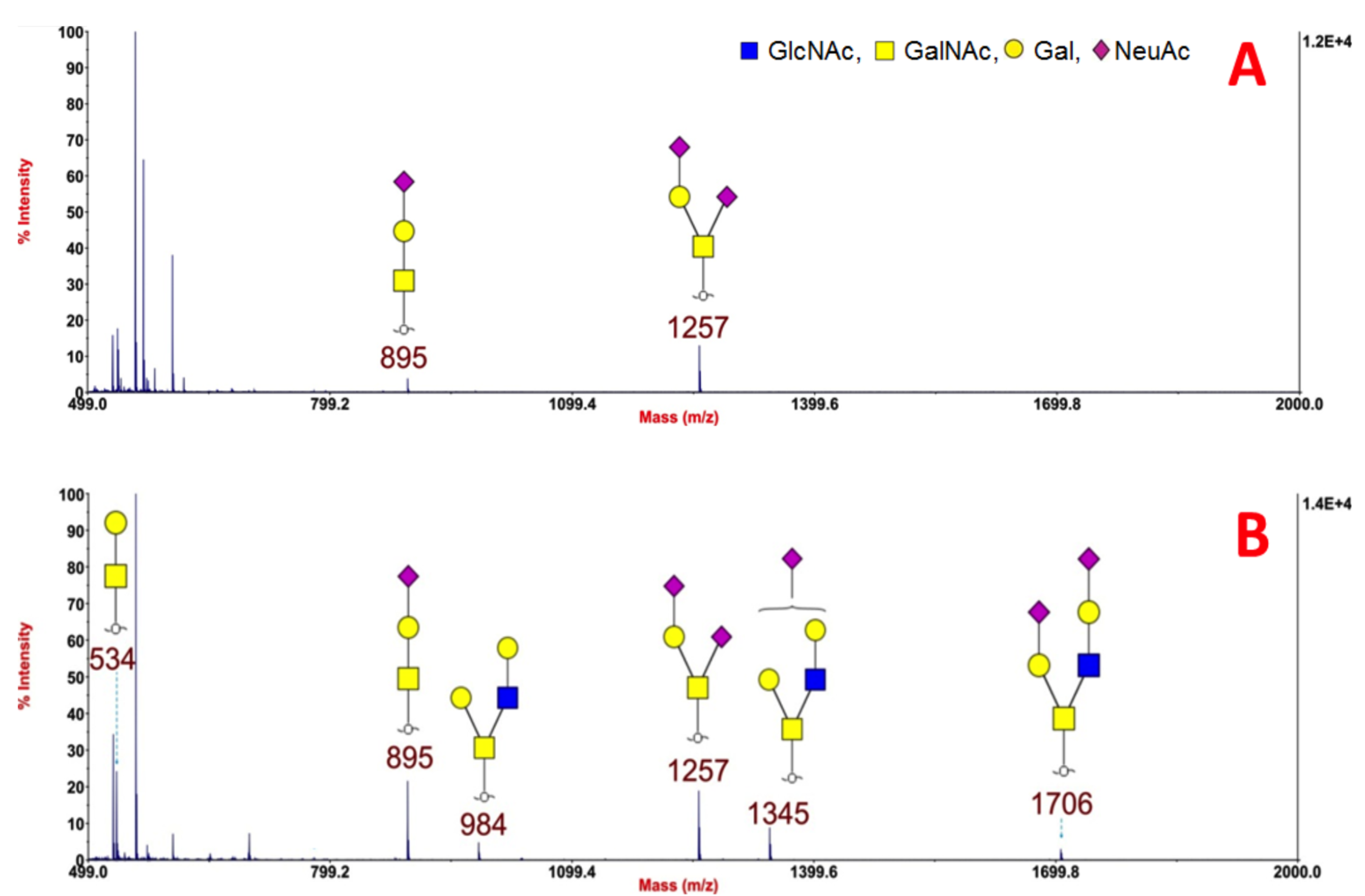

Supplement: S4 Fig — Profiles were obtained from the 35% acetonitrile fraction from a C18 Sep-Pak column. All ions are [M+Na]+. Putative structures are based on the molecular weight, O-glycan biosynthetic pathway and MS/MS data. (TIF) [file pone.0132389.s004.tif]

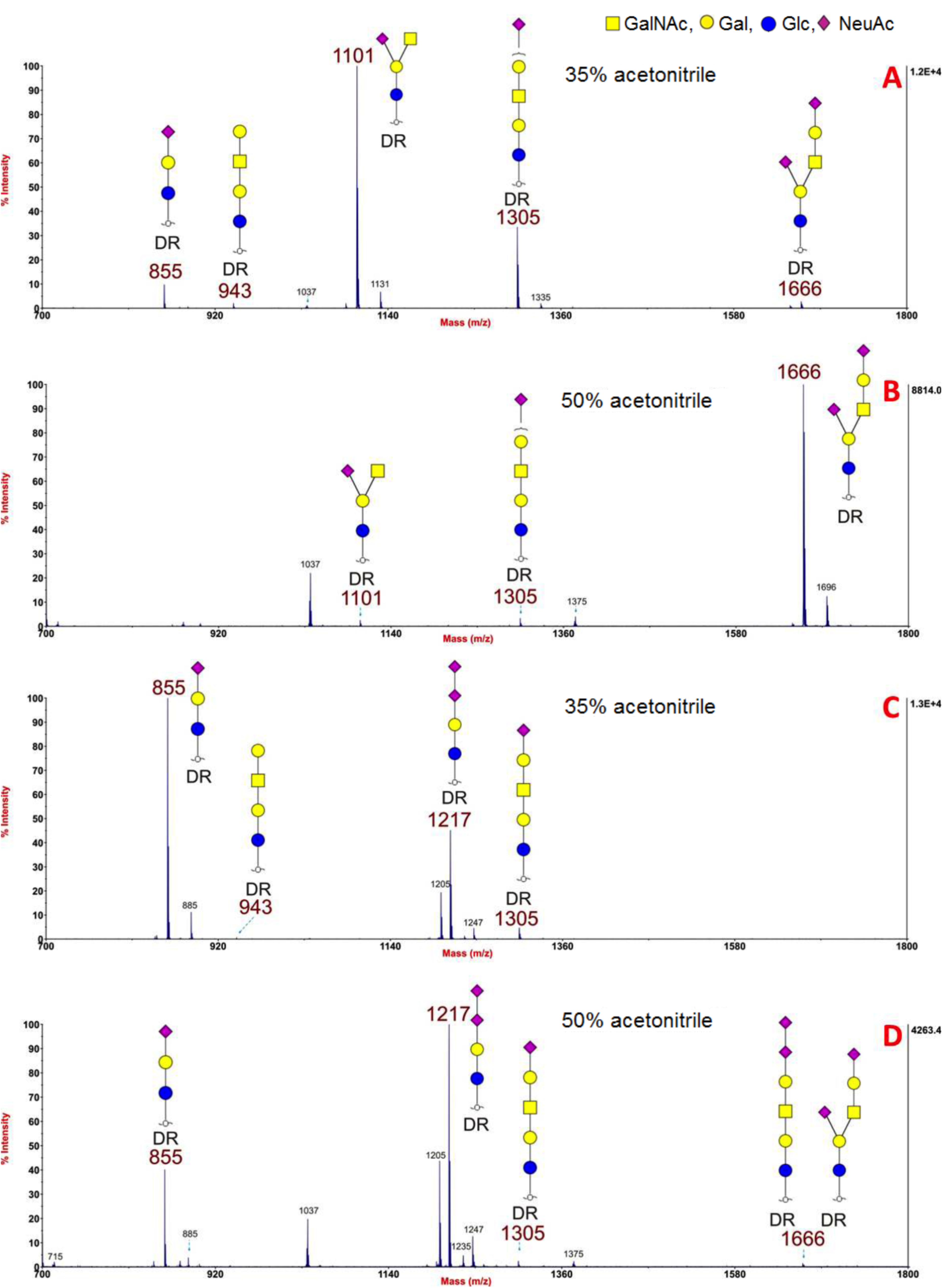

Supplement: S5 Fig — These profiles were obtained from the 35% and 50% acetonitrile fractions from a C18 Sep-Pak column. All ions are [M+Na]+. Putative structures are based on the molecular weight, glycolipid glycan biosynthetic pathway and MS/MS data. (TIF) [file pone.0132389.s005.tif]

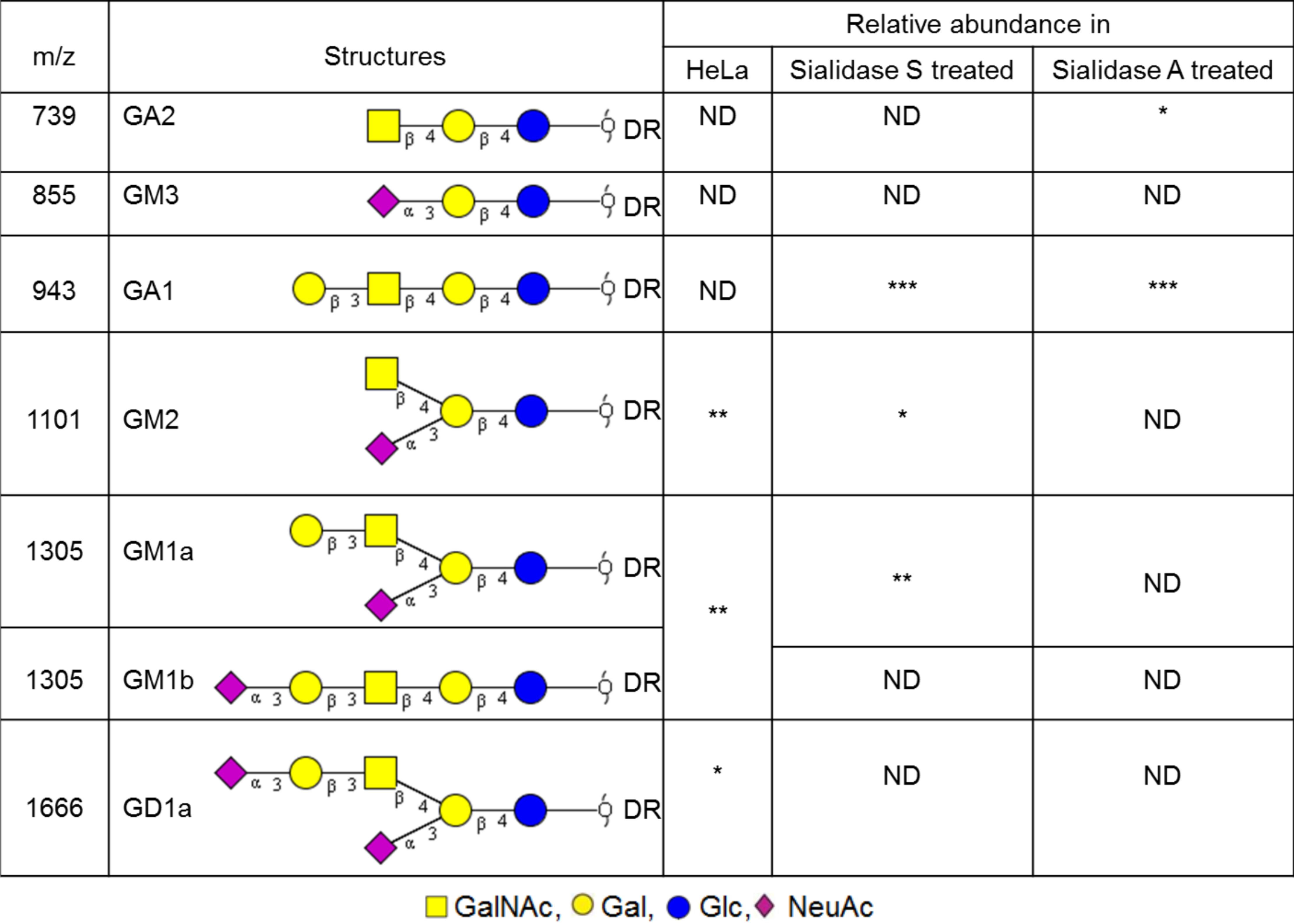

Supplement: S6 Fig — All glycans are deuteroreduced (DR), permethylated and [M+Na]+. Glycan structures are drawn based on molecular weight, glycolipid glycan biosynthetic pathway and MS/MS data. ND, not detected. * = minor (<20%), ** = medium (20–50%), *** = major (>50%). (TIF) [file pone.0132389.s006.tif]
